# Progress in Natural Products Target Discovery Technology

**DOI:** 10.1002/mco2.70777

**Published:** 2026-05-24

**Authors:** Qiyuan Pan, Xiao Yuan, Jinmei Jin, Xin Luan, Cheng Luo, Hongzhuan Chen, Weidong Zhang, Hao Zhang

**Affiliations:** ^1^ Shanghai Frontiers Science Center of TCM Chemical Biology Institute of Interdisciplinary Integrative Medicine Research and Shuguang Hospital Shanghai University of Traditional Chinese Medicine Shanghai China; ^2^ State Key Laboratory of Drug Research Shanghai Institute of Materia Medica Chinese Academy of Sciences Shanghai China; ^3^ State Key Laboratory For Quality Ensurance and Sustainable Use of Dao‐di Herbs Institute of Medicinal Plant Development Chinese Academy of Medical Sciences and Peking Union Medical College Beijing China; ^4^ Department of Phytochemistry School of Pharmacy Second Military Medical University Shanghai China

**Keywords:** artificial intelligence, drug discovery, molecular probes, natural products, target identification

## Abstract

Natural products, owing to their unique biological activities, possess the ability to interact with specific target proteins or regulatory networks, representing a valuable source of innovative drug candidates. However, target identification remains a major bottleneck in natural product‐based drug discovery, largely because of the chemical complexity of natural products and the heterogeneity of biological systems. To address these challenges, various complementary strategies have been developed, including experimental strategies such as chemical proteomics, and computational methods such as artificial intelligence‐driven methods. Nevertheless, reliably advancing a candidate protein hit to a therapeutically relevant and physiologically validated target remains a critical challenge. Focusing on technologies for natural product target discovery, this review systematically summarizes the principles, methodologies, and practical applications of current approaches. Through representative case studies, we further propose a reusable integrated experimental–computational workflow and illustrates how key targets and their modes of action can be identified in real‐world research scenarios. In addition, we discuss common technical and conceptual bottlenecks encountered during target discovery and proposes potential countermeasures. The review provides an actionable reference framework for natural product target identification, with the goal of reducing false‐positive findings and fragmented evidence, thereby improving the robustness of mechanism‐oriented studies and facilitating subsequent translational research.

## Graphical abstract

Here, we provide a comprehensive overview of current technologies and recent advancements in therapeutic target discovery for natural products.

By systematically synthesizing the principles, methodologies, and practical applications of existing experimental and computational strategies, this work provides a more actionable reference framework for natural product target discovery

This synergistic strategy will drive the paradigm shift in next‐generation target identification technologies and accelerate natural product‐based drug discovery.

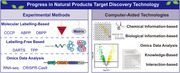

## 1 Introduction

**FIGURE 1:**
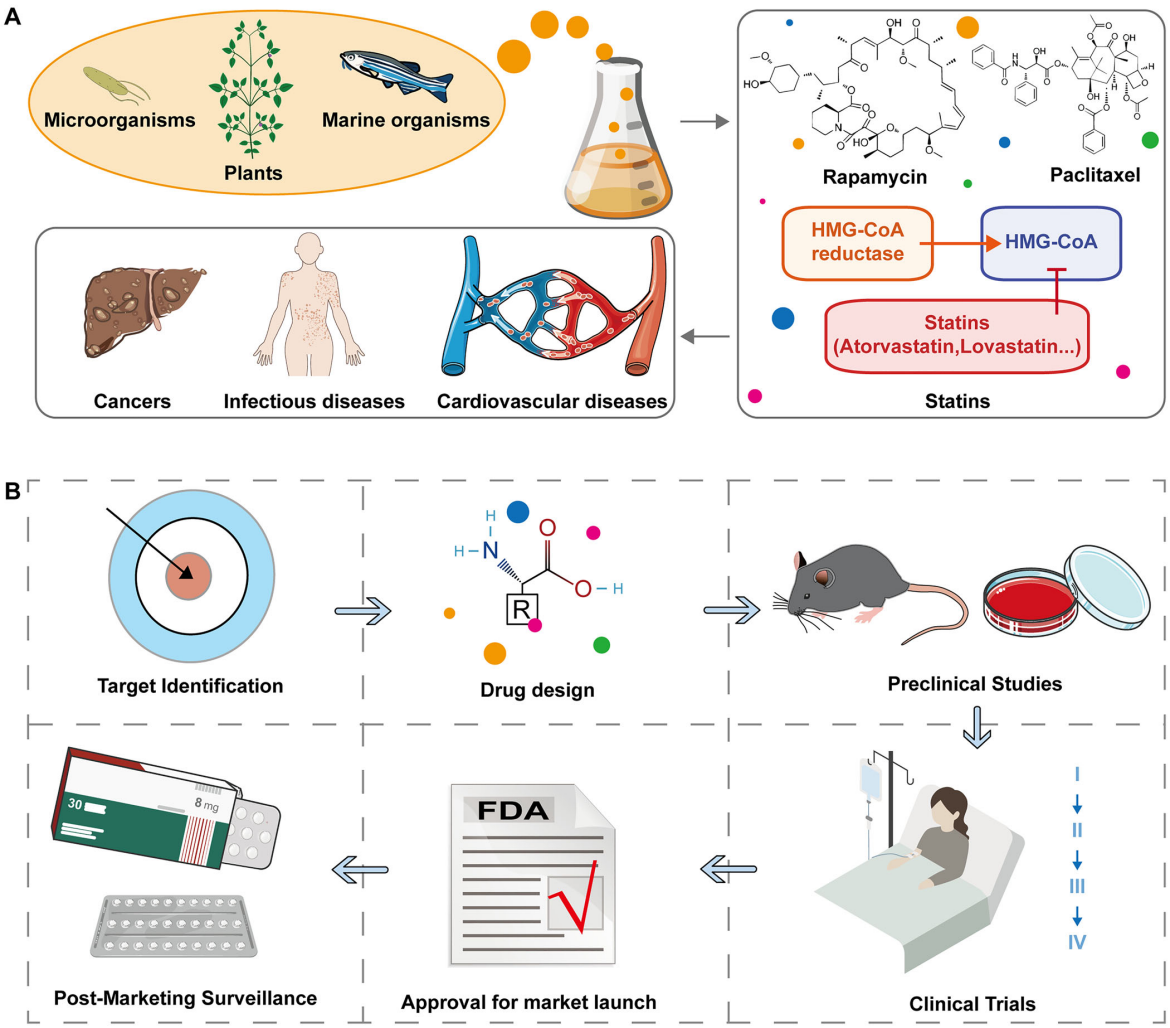
Natural products as a major source of drugs and the central role of target identification in drug development. (A) Natural products (NPs) are derived from diverse biological sources, including microorganisms, plants, and marine organisms, and constitute a major source of clinically approved drugs. Numerous therapeutics used for infectious diseases, cancer, cardiovascular disorders, and metabolic diseases are directly or indirectly derived from natural products, as exemplified by statins, rapamycin, and paclitaxel. (B) Target identification occupies a central role in modern drug development and influences downstream processes, including rational drug design, preclinical evaluation, clinical trials, regulatory approval, and postmarketing surveillance. Abbreviations: HMG‐CoA, 3‐hydroxy‐3‐methylglutaryl‐coenzyme A; FDA, Food and Drug Administration.

Natural products (NPs), typically derived from plants, microorganisms, and marine organisms, have unique chemical structures and distinct bioactivities. They have long served as an important source of lead compounds and therapeutic agents for the treatment of infectious diseases, cancer, hypertension, diabetes, and other indications [1]. The discovery and development of several landmark NP‐derived drugs, include statins [2], rapamycin [3], paclitaxel [4, 5], and anisodamine [6], have provided important paradigms for modern drug development (Figure 1A). Within Traditional Chinese Medicine (TCM) systems, many NPs are initially advanced on the basis of established clinical efficacy, which subsequently drives modern pharmacological investigation and mechanistic elucidation. In recent years, novel NP‐related entities and delivery strategies have continued to emerge. For example, the identification of transient receptor potential vanilloid 1, the receptor for capsaicin, has facilitated the development of new generation of analgesics targeting this receptor [7, 8, 9]. In addition, carbon dots (CDs) with photoluminescent properties and high biocompatibility have been widely explored for optical sensing, bioimaging, and therapeutic applications, particularly through the confinement or delivery of natural active ingredients within nanoscale organic carriers [10, 11, 12]. Carrier‐free nanoparticles, such as nanocrystals, self‐assembled nanoparticles, and extracellular vesicles, have also shown considerable potential for improving the stability, targeting capacity, bioavailability, and pharmacological activity of NPs [13].

In modern drug development, target discovery serves as a critical link between bioactive molecules and disease mechanisms and directly influences subsequent lead optimization, mechanism validation, and indication exploration [14] (Figure 1B). Since the completion of the Human Genome Project in 2003 [15], increasing attention has been devoted to the systematic exploration and validation of therapeutic targets. With advances in systems biology, the understanding of drug action has gradually shifted from the traditional “one drug—one target” paradigm toward a polypharmacological view, in which a single drug may interact with multiple targets and regulatory pathways in the human body [16, 17]. This perspective is particularly relevant to NP research, as many NPs exert biological effects through complex and context‐dependent molecular mechanisms. Whether NP discovery follows a target‐based strategy or a phenotype‐driven approach, defining the relevant molecular targets remains essential for mechanistic interpretation and therapeutic validation [18].

The overall process of drug discovery and development is highly complex and often requires several years to more than a decade to complete [19, 20, 21, 22]. Despite continuous methodological progress, NP target identification is still limited by numerous practical challenges, including long development cycles, high costs, low throughput, and substantial attrition rate. During experimental validation, common limitations include restricted throughput, labor‐intensive workflows, and a high risk of false‐positive or indirect target assignments [23, 24]. In recent years, artificial intelligence (AI), broadly referring to computational systems capable of performing tasks that typically require human intelligence [25], has been increasingly incorporated into multiple stages of the drug discovery pipeline. AI and data‐driven strategies have shown considerable potential in structural optimization, target prediction, biological data integration, and prioritization of candidate targets, thereby improving the efficiency of target discovery [25, 26]. However, computational predictions remain highly dependent on the quality and representativeness of training datasets, are vulnerable to temporal drift and data bias, and often suffer from limited interpretability. Therefore, converting computational associations into experimentally verifiable evidence chains remains a major challenge in NP target discovery and requires systematic methodological integration and standardization [26, 27, 28].

Therefore, this review focuses on NP target discovery as a central scientific issue, systematically summarizing the key challenges and unresolved problems in the identification and validation of NP targets. We then provide a comprehensive review of major experimental and computational strategies currently used for target identification, with particular attention to their underlying principles, applicable scenarios, methodological strengths, and limitations. Furthermore, we discuss recommended validation routes and integrated workflows for improving the reliability of target identification. Finally, by considering emerging technological trends, we highlight future directions for advancing NP target discovery and promoting the translation of mechanistic findings into therapeutic applications [26, 29].

## 2 Conceptual Challenges in NP Target Discovery

Compared with conventional synthetic compounds, target discovery for bioactive NPs remains particularly challenging. These challenges arise not only from methodological and technical limitations but also from the intrinsic structural features of NPs and the complex, often context‐dependent nature of their interactions with biological targets.

### 2.1 Structural Complexity and Chemical Diversity

**FIGURE 2:**
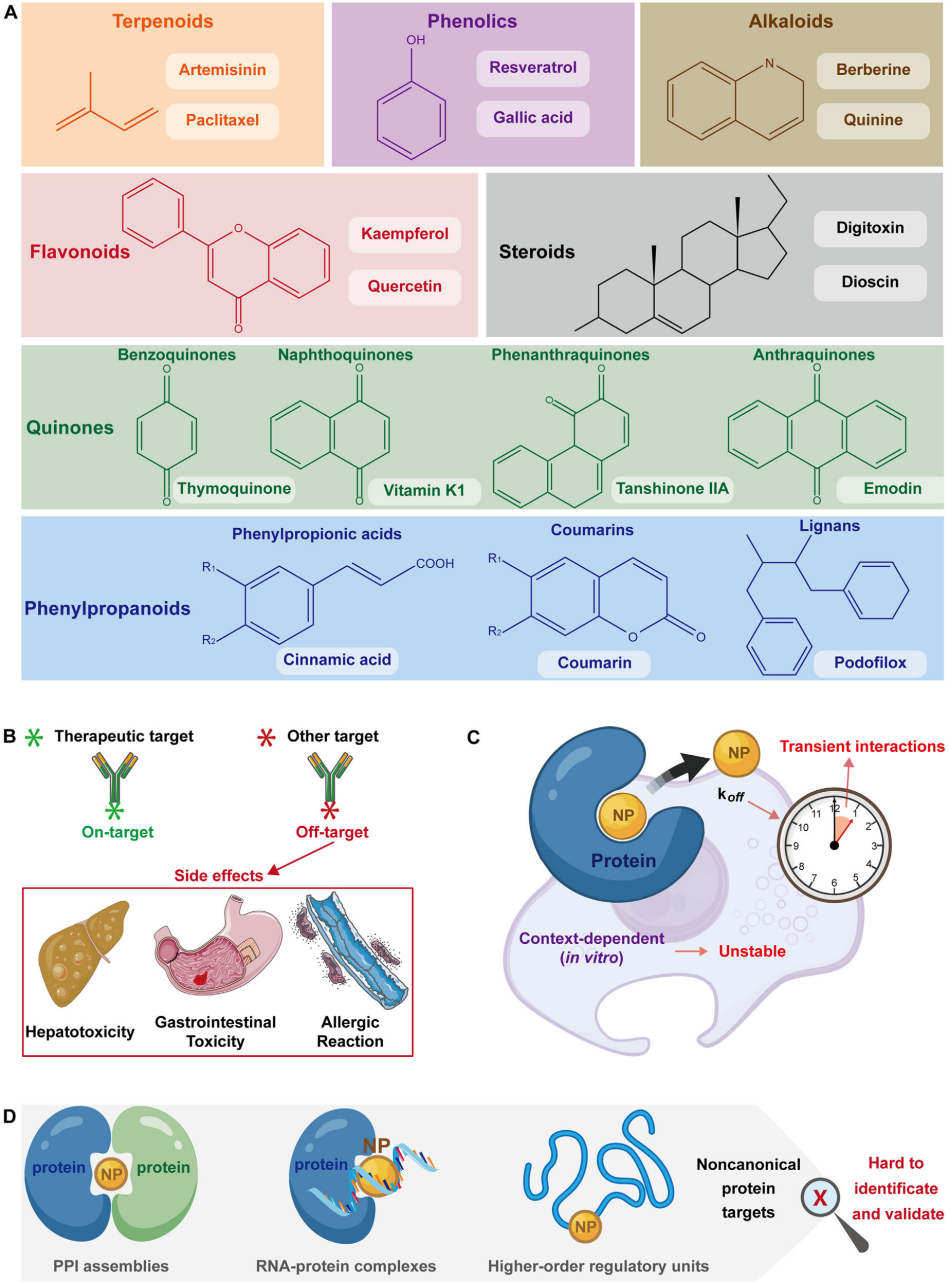
Structural diversity of natural products and major challenges in target identification. (A) Natural products exhibit remarkable structural diversity and can be classified into several major chemical families, including terpenoids, such as artemisinin and paclitaxel; phenolics, such as resveratrol and gallic acid; alkaloids, such as berberine and quinine; flavonoids, such as kaempferol and quercetin; steroids, such as digitoxin and dioscin; quinones, including benzoquinones, naphthoquinones, phenanthraquinones, and anthraquinones, such as thymoquinone, vitamin K1, tanshinone IIA, and emodin; and phenylpropanoids, such as cinnamic acid, coumarin, and podophyllotoxin. This extensive chemical diversity underlies both the broad biological activities of natural products and the complexity of their target identification. (B) Polypharmacological effects of natural products. A single natural product may interact with intended therapeutic targets, referred to as on‐target, as well as unintended proteins, referred to as off‐targets. Off‐target engagement may contribute to adverse effects, including hepatotoxicity, gastrointestinal toxicity, and allergic reactions. (C) Weak, transient, and context‐dependent target engagement. Many natural product–protein interactions are characterized by low binding affinity, rapid dissociation, or strong dependence on cellular context, resulting in unstable interactions that are difficult to capture under conventional in vitro experimental conditions. (D) Noncanonical target modalities. Beyond classical single‐protein targets, natural products may act on protein‐protein interaction (PPI) assemblies, RNA–protein complexes, or higher‐order regulatory units, which are often difficult to identify and validate using conventional target discovery approaches.

NPs often possess complex chemical architectures, including multiple chiral centers, fused ring systems, densely functionalized scaffolds, and diverse stereochemical configurations. These features pose substantial technical challenges for isolation and purification, structural characterization, chemical modification, and pharmacological evaluation [30]. Their structurally complex and multipharmacophore nature enables NPs to engage biological targets through diverse interaction modes and exert unique biological activities. However, these same properties also complicate the elucidation of structure–activity relationships (SARs) and limit the applicability of conventional high‐throughput screening strategies [23, 31, 32] (Figure 2A). When target is known, rational structural modification of NPs can be used to optimize physicochemical properties, improve potency, enhance selectivity, or reduce toxicity, thereby facilitating their development into clinically applicable drugs [32, 33]. However, this target‐guided optimization strategy is often difficult to implement when the target remains unknown. Therefore, accurately localizing therapeutically relevant targets within the highly complex and diverse chemical space of NPs represents a central conceptual and practical challenge in NP target discovery [34].

### 2.2 Polypharmacology and Network‐Level Effects

With the development of systems pharmacology and network biology, accumulating evidence indicates that small‐molecule drugs, particularly NPs, frequently interact with multiple molecular targets simultaneously, giving rise to polypharmacology effects [16, 17]. In this context, therapeutic effects may result from the coordinated modulation of multiple targets, pathways, or regulatory modules rather than from the perturbation of a single dominant node. Mechanistically, pharmacological actions mediated through intended targets are generally defined as on‐target effects, whereas interactions with unintended targets are regarded as off‐target effects. Off‐target activities may contribute to adverse reactions, cumulative toxicity, or unexpected pharmacological outcomes [18] (Figure 2B). Moreover, many diseases are driven by dysregulated networks involving multiple signaling pathways and regulatory nodes, and a single target may participate in distinct pathological states depending on cellular context, tissue type, or disease stage. Thess features further underscore the importance of network‐level mechanisms in drug action. Although the intrinsic multitarget properties of NPs increase the complexity of target discovery and mechanism elucidation [29], they may also confer unique advantages for drug repurposing and systemic therapeutic interventions in multifactorial diseases.

### 2.3 Weak, Transient, or Context‐Dependent Target Interactions

In addition to polypharmacology, the dynamic interaction patterns between NPs and their targets constitute another major challenge. A substantial proportion of NPs may interact with target proteins through weak, transient, low‐affinity, or reversible binding modes. In other cases, target engagement may depend strongly on specific cellular environments, subcellular localization, metabolic states, posttranslational modifications, or disease‐associated physiological conditions [23] (Figure 2C). These characteristics make such interactions difficult to capture and validate reliably under conventional experimental settings. A single target identification method is often insufficient to establish a causal relationship between compound binding and biological activity, owing to the inherent limitations, such as low throughput and a high risk of false positives [23, 24]. Computational and AI‐based methods provide advantages in speed and scalability; however, their predictive performance depends heavily on the quality, representativeness, and completeness of available datasets and protein structural information [26]. Reliable target prediction and validation often require evidence of structural complementarity and experimentally supported target engagement. Therefore, the absence of resolved target structures, annotated binding pockets, or reliable interaction data remains a major bottleneck that limits both computational prediction and downstream experimental validation [26, 28].

### 2.4 Nonprotein and Unconventional Targets

Traditional target discovery has largely focused on proteins with well‐defined enzymatic activities, receptor functions, or ligand‐binding pockets. However, increasing evidence suggests that the molecular targets of NPs are far more diverse, encompassing not only proteins but also nucleic acids, peptides, lipids, metabolites, and biomolecular complexes [35] (Figure 2D). This expanded target spectrum introduces additional challenges for the identification and validation of unconventional targets, particularly those lacking stable conformations, canonical binding pockets, or easily measurable biochemical activities [36]. In addition, certain chemoproteomics strategies require exogenous labeling, probe synthesis, or chemical derivatization. These modifications may disrupt the intrinsic bioactivity of NPs, thereby complicating the accurate identification of unconventional targets [14]. Furthermore, NPs may exert biological effects indirectly by modulating the stability, formation, or dissociation of biomolecular complexes. For instance, regulation of RNA–protein interactions or other macromolecular assemblies can produce downstream pharmacological effects without requiring direct inhibition or activation of a classical protein target. These findings broaden the traditional concept of druggable targets and highlight the need for target discovery strategies capable of capturing noncanonical, dynamic, and context‐dependent mechanisms of action (MOA) [37].

## 3 Experimental Strategies for NP Target Identification

Experimental strategies provide direct evidence for determining whether NP treatment induces characteristic phenotypic changes in in vitro and in vivo models, and whether these effects are mediated through specific molecular targets by altering their activity, stability, expression, or interaction networks. At present, chemical proteomics represents one of the most widely used approaches in NP target discovery. This approach encompasses multiple strategies, including probe‐based labeling, affinity enrichment, stability‐based assays, quantitative proteomics, and related target deconvolution techniques. In addition, multiomics technologies, including transcriptomics, genomics, proteomics, and metabolomics, facilitate the identification of signaling pathways and functional networks associated with drug action, thereby enabling deeper elucidation of the MOA linking NPs to their biological targets (Table 1).

**TABLE 1:** Label‐based and label‐free experimental techniques.

Techniques	Binding mode	Throughput and cost	Outcome quality	Application scope
CCCP [18]	Noncovalent	High‐throughput; time‐consuming	Moderate accuracy; high false positive rate	Noncovalent NPs; unbiased targets
ABPP [38]	Covalent	High‐throughput; time‐consuming	High (covalent) or moderate (photoaffinity) accuracy	Activity‐specific NPs; enzyme/protein families
DBPP [39]	Noncovalent	Mid‐to‐high throughput; time‐efficient	Moderate accuracy; reduced false positive rate	Degrader‐enabled NPs; multitarget identification with PROTAC
DARTS [40]	Noncovalent	Low‐to‐mid throughput; time‐efficient	Moderate accuracy; limited sensitivity	Unmodified NPs or extracts; protease
TPP [41]	Noncovalent	High‐throughput; time‐consuming	High accuracy	Thermostability‐modulating NPs; proteome‐wide target
SPROX [42]	Noncovalent	Low throughput; time‐consuming	Moderate accuracy; limited coverage	Unmodified NPs; met‐containing proteins

Abbreviations: ABPP, activity‐based protein profiling; CCCP, compound‐centric chemical proteomics; DARTS, drug affinity responsive target stability; DBPP, degradation‐based protein profiling; SPROX; stability of proteins from rates of oxidation; TPP, thermal proteome profiling.

### 3.1 Affinity‐ and Label‐Based Approaches

**FIGURE 3:**
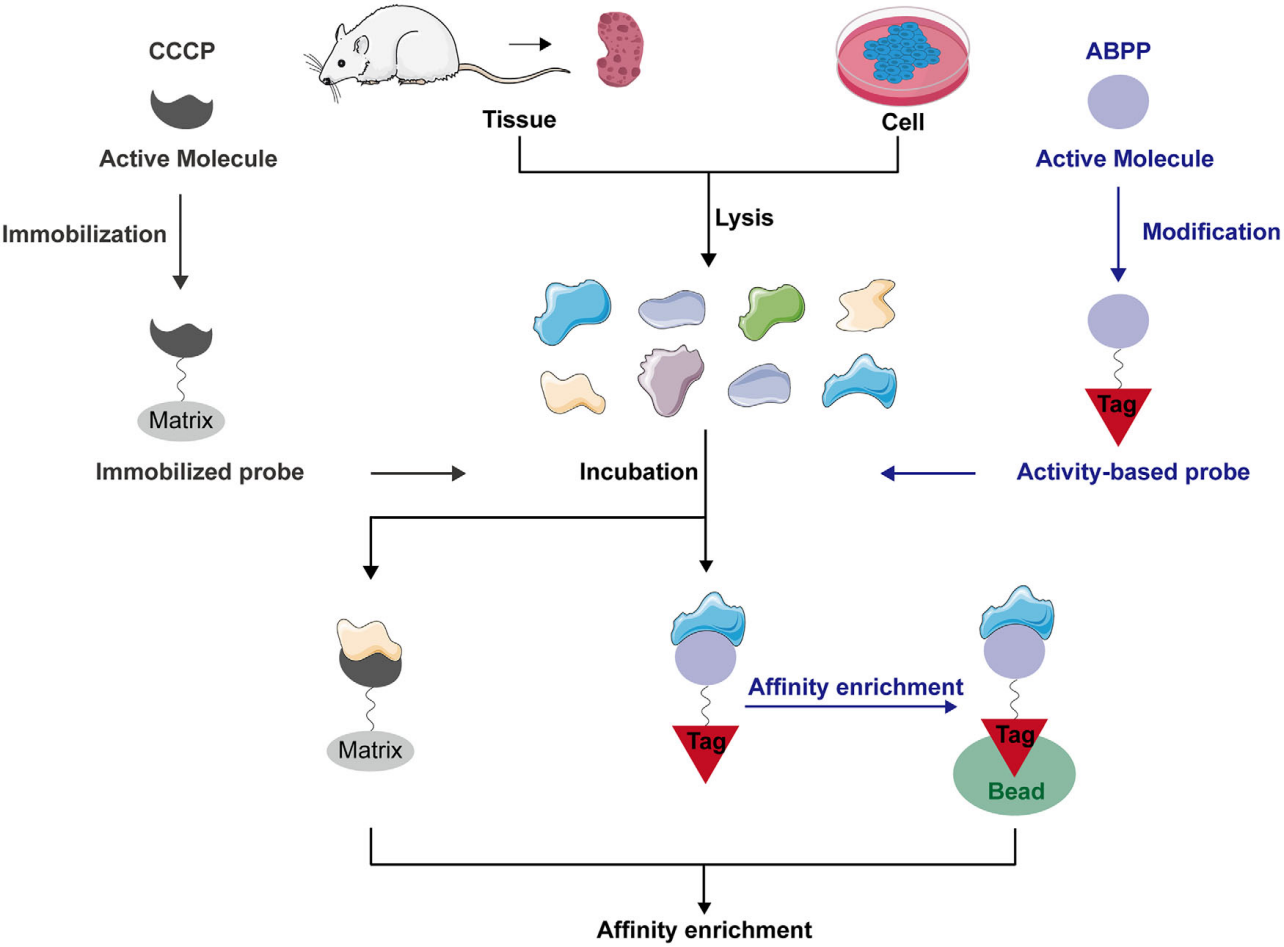
Molecular probe‐based strategies for natural product target identification. Depending on the type of probe employed, molecular probe‐based target identification strategies can be broadly classified into two major approaches: affinity‐based chemical proteomics, also known as compound‐centric chemical proteomics (CCCP), and activity‐based protein profiling (ABPP). The left panel illustrates CCCP, in which natural products are immobilized on solid supports to enrich interacting proteins. The right panel depicts ABPP, where natural products are chemically modified to incorporate detectable tags for capturing target proteins.

Label‐based target identification methods are important tools for discovering NP targets [34]. These approaches generally combine chemical proteomics [18] with affinity enrichment or affinity chromatography‐based techniques. The workflow can be divided into two major steps. First, a functional probe is designed and synthesized by introducing an affinity tag, photo‐crosslinking group, or other reactive moiety into the NP scaffold. Second, the probe is used to capture or enrich interacting proteins, followed by protein identification, quantitative analysis, and functional validation [14]. Common labeling techniques include biotin labeling [43], photoaffinity labeling (PAL) [34], and bioorthogonal reaction labeling [44]. According to probe design and mechanism of action, probe‐dependent target discovery methods can generally be classified into affinity‐based and activity‐based approaches [34] (Figure 3).

#### 3.1.1 Compound‐Centric Chemical Proteomics

Compound‐centric chemical proteomics (CCCP), also referred to as affinity‐based chemical proteomics, originated from affinity chromatography [45] and has evolved through the integration of modern mass spectrometry (MS)‐based proteomics with classical affinity enrichment strategies [46]. Driven by advances in high‐resolution MS, CCCP has become a robust and widely used method for NP target discovery at the protein level [18]. Typical CCCP workflow involves modifying NPs with molecular handle or affinity tag, immobilizing onto an inert solid‐phase matrix, such as agarose beads. The immobilized compound is employed in then incubated with cell or tissue lysates under bioactivity‐preserving conditions. After target binding, nonspecific proteins are removed through washing, while specifically bound proteins are eluted by competitive displacement or other elution strategies. The enriched proteins are subsequently digested with trypsin and analyzed by liquid chromatography–tandem MS (LC–MS/MS) for target identification [18, 46].

Unlike strategies relying on catalytic site labeling, CCCP does not require targets to possess enzymatically active residues amenable to covalent probe modification. Therefore, it can theoretically cover a broader range of target classes, including nonenzymatic proteins, scaffold proteins, and protein complexes, making it particularly suitable for discovering novel or unexpected targets [18]. Another key advantage of CCCP is its compatibility with relatively physiological sample contexts, as it can be applied to diverse biological materials, including cells, tissues, and species‐specific samples. This feature allows better simulating disease‐relevant molecular environments. However, CCCP cannot directly distinguish the functional activation state of target proteins and may enrich indirect or nonspecific binders, thereby increasing the risk of false‐positive target assignments [14].

#### 3.1.2 Activity‐Based Protein Profiling

Activity‐based protein profiling (ABPP) is an important chemical proteomics strategy that uses small‐molecule activity‐based probes (ABPs) to investigate the functional states of enzymes in complex biological systems, particularly their catalytic activities under physiological or disease conditions [38, 47, 48]. Accordingly, ABPP has been widely applied in studies of inflammation [49], malaria [50], cancer [51], and other disease areas. Small‐molecule ABPs generally consist of three components: a reactive group, a linker, and a reporter tag. The reactive group covalently interacts with specific active‐site residues or functional groups within target proteins or protein families. ABPP can help uncover allosteric sites, representing a comparative advantage over certain conventional target discovery approaches [52]. The linker connects the reactive group to the reporter tag and can be optimized to improve probe stability, permeability, specificity, or binding performance [46]. The reporter tag enables visualization, affinity enrichment, separation, or quantitative analysis of probe‐labeled proteins. Affinity‐based probes (AfBPs), particularly photoaffinity probes, can covalently capture biomolecules after activation and are especially useful for studying NPs that bind reversibly to potential targets [53, 54, 55]. In existing NP target discovery studies, ABPP‐related techniques have been combined with various labeling strategies, including biotin labeling [43], bioorthogonal reaction‐based labeling, and PAL [54, 56].

*Biotin Labeling Method*. NP targets can be identified by synthesizing chemical probes or derivatives of bioactive NPs [57]. Biotin, also known as vitamin H, exhibits strong and specific binding to avidin or streptavidin and is therefore one of the most widely used affinity tags for target enrichment in ABPP and related chemical proteomics workflows [47]. Biotin labeling has been extensively used for NP target identification. Generally, biotin‐modified NP probes can interact with target proteins in cells or lysates before affinity purification, making this strategy practical and widely adopted [58, 59]. However, biotin labeling often requires chemical modification of the parent compound, which may limit its applicability [39]. Additionally, the relatively bulky biotin tag may alter the physicochemical properties, cellular distribution, binding specificity, or biological activity of NP probes, thereby reducing target specificity and confidence [34].

*Reactive Labeling Method*. Click chemistry enables the modular connection of small chemical units through highly selective reactions, typically forming new carbon‐heteroatom linkages [60]. A series of bioorthogonal reactions have been developed and applied in targeted drug discovery, particularly in anticancer drugs research [44, 61]. These reactions can also be incorporated into molecular probe design for NP target identification. A major advantage of reactive labeling methods is that they often require only small bioorthogonal handles, such as azide or alkyne groups, rather than large reporter tags such as biotin [34]. This simplicity makes it one of the most mainstream methods for target identification. Owing to their high efficiency, specificity, and compatibility with biological systems, click chemistry‐ and bioorthogonal reactions‐based probes have substantially advanced ABPP and play important roles in NP target discovery [62, 63].

*PAL Method*. Bioactive NPs can also be converted into photoaffinity probes that covalently capture target proteins upon light activation [64]. When NPs lack intrinsic covalent reactivity but interact with targets through noncovalent forces, such as hydrogen bonding, hydrophobic interactions, or electrostatic interactions, PAL enables covalent stabilization and subsequent identification of these otherwise reversible interactions [14]. Common photo‐reactive functional groups include benzophenones, diazirines, and aryl azides [65, 66, 67]. Probes containing a photoactivatable group attached to a NP scaffold are often referred to as AfBP or photoaffinity probes [54, 68]. Upon photoactivation like for example by ultraviolet (UV) irradiation, NP‐derived photoaffinity probes form covalent crosslinks with proximal target proteins in live cells or lysates [34]. The resulting covalent complexes can then be enriched and detected, often through click chemistry‐mediated reporter conjugation, thereby improving the sensitivity of target identification [69, 70].

#### 3.1.3 Degradation‐Based Protein Profiling

To facilitate the identification of multiple targets of NPs, degradation‐based protein profiling (DBPP) has been proposed as a novel target discovery strategy [39]. DBPP combines emerging proteolysis targeting chimeras (PROTAC) technology [71] with quantitative proteomics and immunoprecipitation–MS (IP–MS) for target identification. In this approach, candidate targets are inferred from proteins that show compound‐dependent degradation or relative downregulation in quantitative proteomic datasets. Whereas ABPP primarily focuses on the direct interactions between small‐molecule probes and target proteins, DBPP exploits chemical‐induced protein‐protein interactions (PPIs) that recruit target proteins to degradation machinery. In theory, this strategy can reveal targets that interact with NP‐derived ligands with moderate or even weak binding affinity [39]. Therefore, DBPP represents a powerful complement to conventional chemical proteomics techniques and may accelerate both drug target discovery and the development of new drugs based on NP scaffolds.

### 3.2 Label‐Free and Biophysical Methods

Although label‐based approaches have substantially advanced NP target identification, the introduction of exogenous tags, linkers, or reactive groups may alter the native structure, bioactivity, cellular permeability, or target‐binding profile of NPs. These modifications can compromise the accuracy of target identification and limit the applicability of probe‐dependent strategies [14]. To address these limitations, label‐free chemical proteomics and biophysical approaches have been developed to detect target engagement without requiring structural modification of the parent compound.

#### 3.2.1 Drug Affinity Responsive Target Stability

**FIGURE 4:**
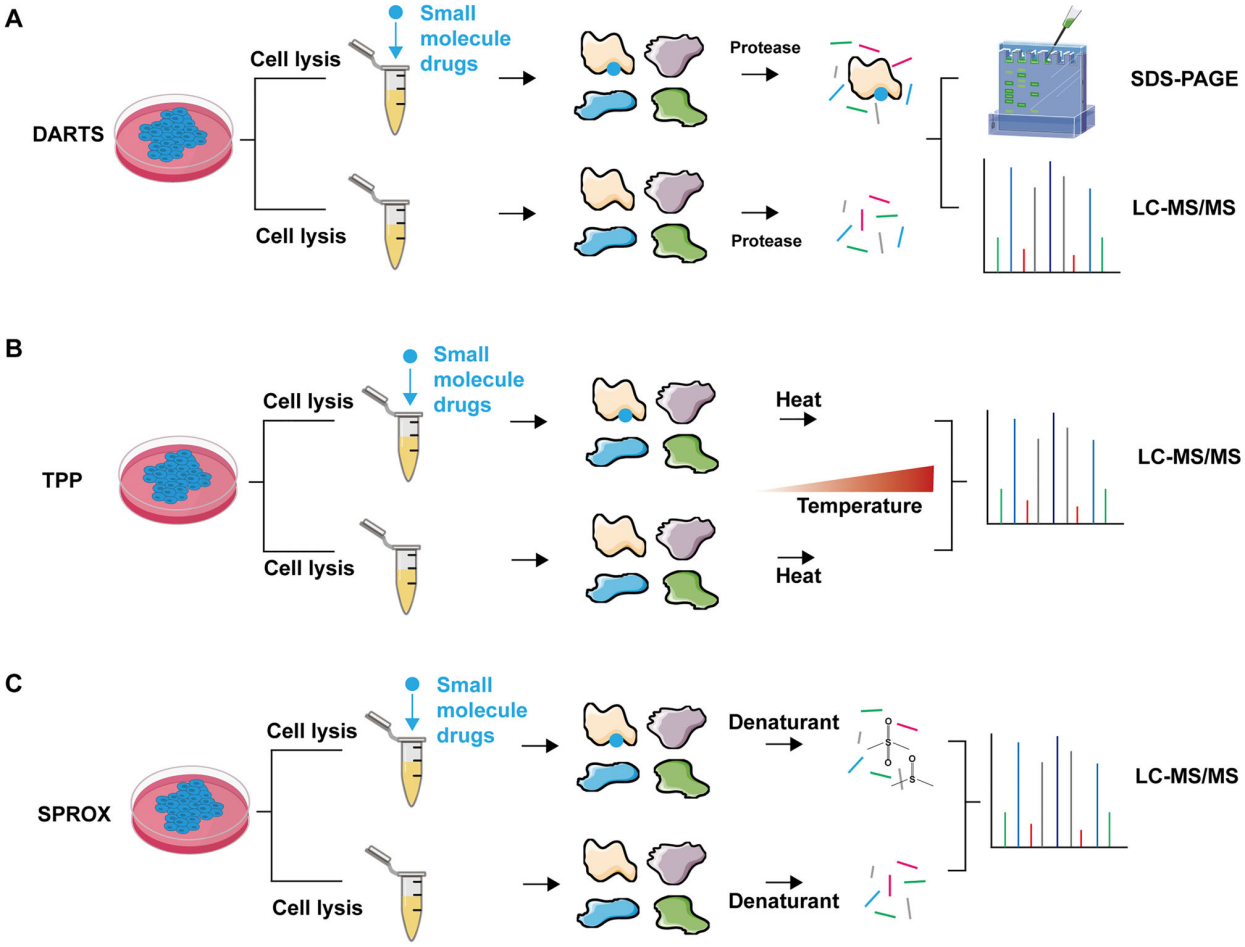
Label‐free and biophysical methods for natural product target identification. (A) DARTS (Drug Affinity Responsive Target Stability): ligand binding alters the susceptibility of target proteins to proteolytic digestion, and differential proteolysis patterns are analyzed by sodium dodecyl sulfate‐polyacrylamide gel electrophoresis (SDS‐PAGE) or liquid chromatography–tandem mass spectrometry (LC–MS/MS) to infer potential targets. (B) TPP/CETSA (thermal proteome profiling/cellular thermal shift assay): ligand binding alters protein thermal stability, which is assessed across temperature gradients by measuring soluble protein fractions followed by quantitative LC–MS/MS analysis. (C) SPROX (stability of proteins from rates of oxidation): hydrogen peroxide‐mediated oxidation is used to quantify methionine oxidation, enabling evaluation of the thermodynamic stability of proteins and protein–ligand complexes.

Drug affinity responsive target stability (DARTS) is one of the most commonly used label‐free techniques for identifying direct drug targets. DARTS is based on the principle that ligand binding can alter the conformational stability of target proteins and reduce their susceptibility to protease‐mediated digestion [40] (Figure 4A). One major advantage of DARTS is that it does not require structural modification of NPs, thereby preserving the native structure and activity of the compound. Moreover, DARTS is particularly suitable for complex protein samples, such as whole‐cell lysates [72]. In a typical DARTS experiment, protein samples are incubated with a NP and then subjected to limited proteolysis. Proteins stabilized by ligand binding are relatively protected from degradation and can be detected by immunoblotting or MS‐based proteomics. When DARTS is applied for unbiased target discovery, it is usually coupled with quantitative proteomic analysis after proteolysis to identify proteins showing altered protease sensitivity [73]. DARTS can also be combined with other target discovery strategies to provide orthogonal evidence supporting candidate targets identified by affinity‐based, activity‐based, or computational approaches [70, 74, 75].

#### 3.2.2 Thermal Proteome Profiling

The cellular thermal shift assay (CETSA) was developed to assess intracellular target engagement by measuring ligand‐induced changes in protein thermal stability [76]. CETSA can be performed in both intact cells and cell lysates, making it more physiologically relevant than many other label‐free methods [77]. CETSA is based on the principle that ligands binding can alter, often increase, the thermal stability of proteins [76]. A standard CETSA workflow involves treating cells or lysates with a candidate compound, exposing samples to a temperature gradient to induce protein denaturation, separating soluble proteins from precipitated fractions, and detecting the abundance of target protein remaining in the soluble fraction [78]. Ligand‐bound proteins are frequently protected from heat‐induced denaturation and precipitation, thereby remaining soluble. However, CETSA may not be suitable for proteins with highly heterogeneous or those whose thermal stability behavior or for targets whose thermal stability is not measurably altered upon ligand binding [79]. Classic CETSA technique commonly uses Western blotting as the detection method and is therefore particularly useful for validating predefined candidate targets [80, 81].

Thermal proteome profiling (TPP), also known as MS‐CETSA, is derived from CETSA and combines thermal stability measurement with quantitative MS (Figure 4B). By incorporating proteome‐wide quantitative MS analysis, TPP overcomes the limited sensitivity and throughput of conventional CETSA and enables unbiased measurement of protein thermal stability across the proteome, including in intact cells [34, 41]. TPP can identify both direct targets and indirect downstream proteins affected by compound treatment. TPP and its modified versions have been widely used in NP target identification [77, 82]. However, high experimental cost, complex sample processing, and demanding data analysis remain important challenges for the broader application of TPP.

#### 3.2.3 Stability of Proteins From Rates of Oxidation

Stability of proteins from rates of oxidation (SPROX) is a label‐free method compatible with quantitative chemical proteomics. SPROX uses hydrogen peroxide‐mediated oxidation to monitor ligand‐induced changes in protein thermodynamic stability, typically by measuring the oxidation levels of methionine residues [42, 83, 84] (Figure 4C). Upon ligand binding, changes in protein folding or stability can alter the exposure of methionine residues to oxidation, thereby providing information about protein–ligand interactions. However, it depends on the presence and detectable oxidation of methionine residues that report stability changes upon ligand binding. In addition, SPROX often requires relatively high protein concentrations and careful optimization of oxidation conditions, which may restrict its applicability to low‐abundance proteins or complex biological samples [79].

### 3.3 Omics‐Driven Experimental Approaches

Next‐generation sequencing has become a powerful tool for transcriptome analysis [85, 86]. RNA sequencing (RNA‐seq) enables quantitative profiling of transcriptomic changes before and after drug treatment in cells, tissues, or disease models [87, 88, 89]. Single‐cell RNA‐seq (scRNA‐seq) further allows the analysis of gene expression heterogeneity, identification of rare cell populations, and characterization of dynamic cellular states in complex disease contexts [90]. In cancer research, for example, scRNA‐seq enables detailed characterization of the tumor microenvironment (TME) and can help identify key regulatory signals of drugs or drug‐responsive pathways within specific cell subpopulations [91, 92, 93, 94].

**FIGURE 5:**
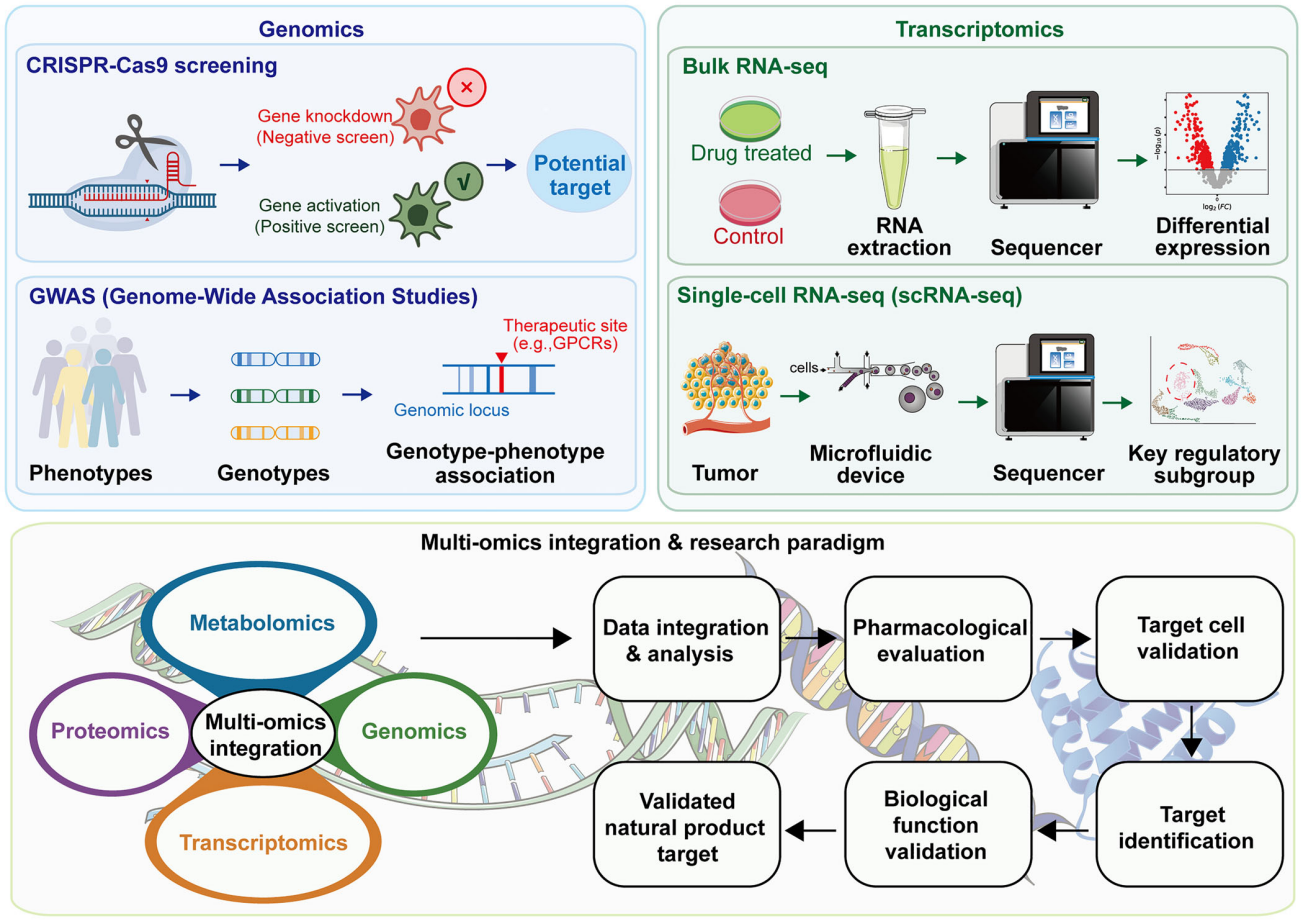
Multiomics‐based strategies for natural product target discovery and validation. Multiple omics technologies have been widely applied to target identification, including clustered regularly interspaced short palindromic repeats associated protein 9 (CRISPR–Cas9) screening, genome‐wide association studies (GWAS), bulk RNA sequencing (RNA‐seq), and single‐cell RNA sequencing (scRNA‐seq). CRISPR–Cas9 screening enables positive or negative selection to identify genes mediating drug responses. Accelerating natural product target discovery requires the integration of multiomics methods, combining genomic, transcriptomic, proteomic, and metabolomic data with pharmacological evaluation, target cell identification, target validation, and functional characterization to achieve robust target confirmation.

Genomics‐based approaches also provide important support for target discovery. Genome‐scale clustered regularly interspaced short palindromic repeats associated protein 9 (CRISPR–Cas9) screening enables systematic gene perturbation at specific genomic loc and supports both negative and positive selection screening in human cells. By observing how gene knockout, knockdown, or activation affects drug sensitivity or cellular phenotypes, CRISPR‐based approaches can reveal genes and pathways functionally associated with NP activity [95] (Figure 5). Genome‐wide association studies (GWASs) can identify disease‐associated genetic loci and potential therapeutic targets through genotype–phenotype association analysis, thereby providing clues for the target discovery of NPs in various disease contexts [96, 97, 98]. Metabolomics, supported by analytical platforms such as MS and nuclear magnetic resonance [99] spectroscopy, enables systematic profiling of metabolites involved in cellular metabolism. By comparing metabolic changes induced by NP treatment, metabolomics can be used to identify biomarkers, reveal perturbed metabolic pathways, clarify MOA, and prioritize potential targets in diverse human diseases [100, 101].

With the rapid development of genomics, transcriptomics, proteomics, and metabolomics, multiomics integration has become an increasingly effective strategy for target discovery. The emergence of single‐cell and spatial transcriptomics provides new perspectives for NP target discovery and mechanism elucidation, showing strong potential in studies of inflammatory diseases [102], cancer [103], diabetic nephropathy [104], and other conditions. Integrating spatial transcriptomics with other omics technologies facilitates the identification of disease‐relevant cell subpopulations, enables more precise characterization of cell–cell interactions, and maps spatially organized communication networks within tissues. Such integration can support more detailed exploration systems‐level pharmacological effects of NPs [29, 105, 106]. Based on these advances, an integrated research paradigm of “pharmacological evaluation‐target cell confirmation‐target identification‐biological function validation” has been proposed. This framework provides an operational strategy for the target validation of NPs and other small‐molecule compounds and may improve the reliability and translational relevance of target discovery studies [29].

## 4 Computational and AI‐Based Target Discovery Technologies

Accurately identifying drug targets from thousands of potential candidate proteins remains challenging when relying solely on traditional experimental approaches. Computational methods can substantially narrow the candidate target space. Owing to their advantages in reducing labor intensity, experimental cost, and time requirements, target discovery algorithms have been widely applied in drug discovery research [107]. AI refers to the capability of machines to perform tasks that typically require human intelligence [25]. With the support of AI‐based tools, multiple stages of drug discovery, including target prediction, compound optimization, and mechanism interpretation, can be significantly accelerated. In the context of NP target discovery, computational technologies can be broadly classified into four categories according to their underlying algorithmic principles.

### 4.1 Ligand‐Based Target Prediction

**FIGURE 6:**
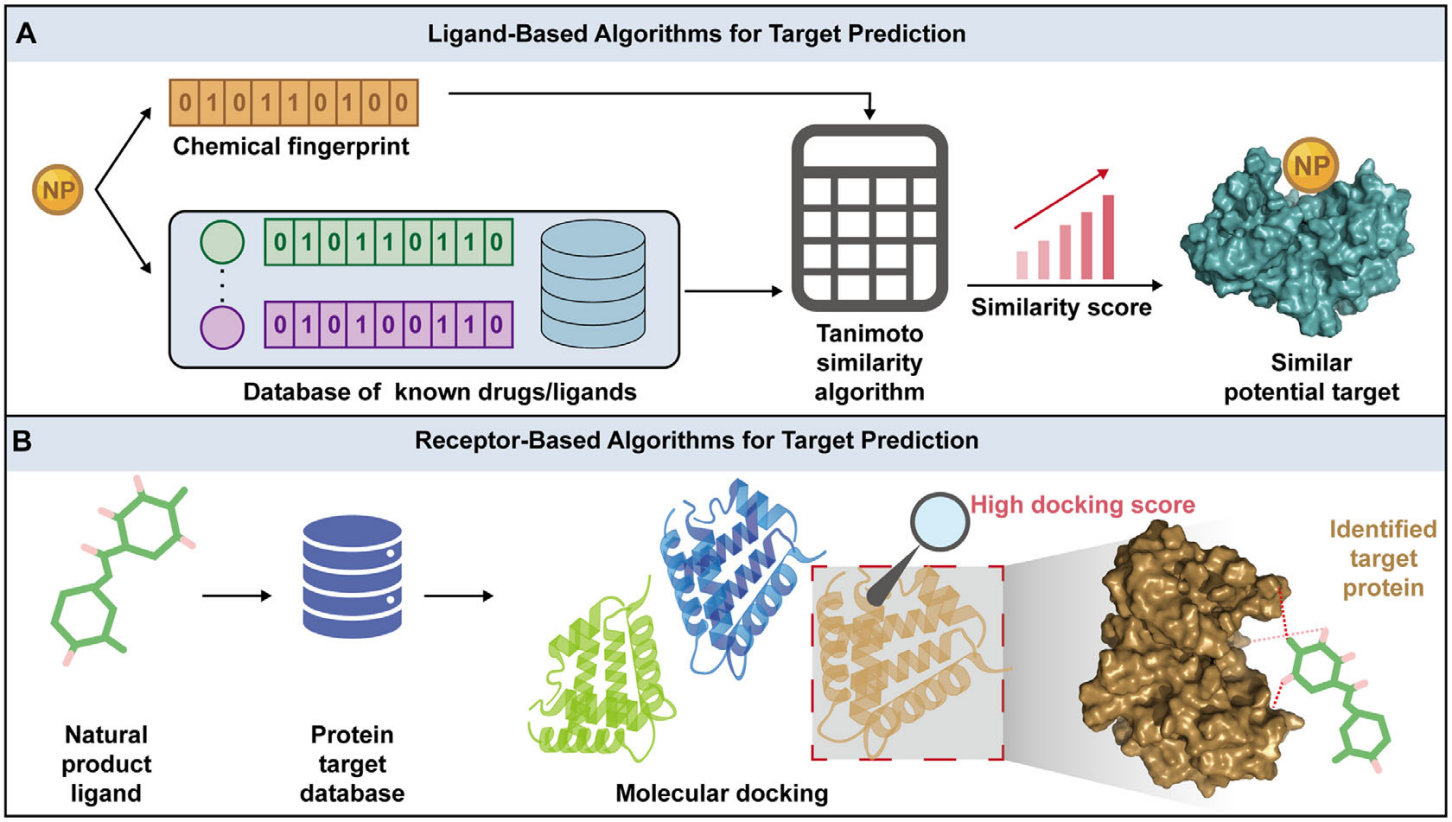
Computational strategies for natural product target prediction. (A) Ligand‐based algorithms for target prediction. Natural products are encoded as chemical fingerprints and compared with known ligands or drug databases using similarity metrics, such as the Tanimoto coefficient, to prioritize candidate targets. (B) Receptor‐based algorithms for target prediction. Molecular docking and reverse docking rank potential targets and predict binding modes based on three‐dimensional protein structures.

Chemical similarity is a fundamental concept in chemoinformatics and provides an important basis for computational target prediction. Previous studies have shown that compounds with similar chemical structures often exhibit similar biological activities [108]. Therefore, by evaluating whether NPs share structural similarities with known bioactive compounds, it is possible to infer their potential targets [109]. The target prediction methods based on chemoinformatics and chemical similarity generally represent small molecule using chemical fingerprints and apply similarity algorithms, such as the Tanimoto coefficient, to quantify the similarity between two compounds [110]. Because many drugs can interact with multiple targets, ligand‐based prediction can also help distinguish desired therapeutic efficacy from potential off‐target effects or adverse reactions [107, 111]. Therefore, exploring drug–target binding patterns and predicting targets based on ligand structural similarity represent feasible strategies for NP target discovery [111] (Figure 6A and Table 2).

**TABLE 2:** Computational tools based on ligand and receptor structures.

Principle	Tool name	Data source	License type	Availability status	Latest version	Website
Comparative analysis based on ligand structural features	TarPred [112]	CTD	Not specified	Web server	2015	http://www.dddc.ac.cn/tarpred
	TargetHunter [113]	ChEMBL	Not specified	Web server	2016	http://www.dddc.ac.cn/tarpred
	MolTarPred [114]	ChEMBL	Open‐source	Web server	2019	http://moltarpred.marseille.inserm.fr/
	ChemMapper [115]	ChEMBL, DrugBank, BindingDB, KEGG, PDB; UniProt, GO, ZINC	Open‐source	Web server	2013	http://lilab.ecust.edu.cn/chemmapper/
	SwissTargetPreDiction [116, 117]	ChEMBL	Free	Web server	2019	www.swisstargetprediction.ch
	SEA [118]	ChEMBL, MDDR	Free‐of‐charge	Web server	2007	https://sea.bkslab.org/
	LigTmap [119]	PDB‐bind	Free	Web server	2021	https://cbbio.online/LigTMap
	Fragment‐based method [120, 121]	DNP, COBRA collection	Publisher copyright	Not released	2014	——
Reverse screening based on ligand–protein structure	TarFisDock [122]	PDTD	Free‐to‐use via registration	Web server	2006	http://www.dddc.ac.cn/tarfisdock/
	DRAR‐CPI [123]	UniProt, DrugBank	Free	Web server	2011	http://cpi.bio‐x.cn/drar/
	IDTarget [124]	PDB	Free	Web server	2012	http://idtarget.rcas.sinica.edu.tw
	GalaxySagittarius_AF [125]	PDB, AFDB	Free	Web server	2024	https://galaxy.seoklab.org/sagittarius_af
	ACID [126]	PDB‐bind, DrugBank, Uniprot, PDB	Free	Web server	2019	http://chemyang.ccnu.edu.cn/ccb/server/ACID
	PharmMapper [127, 128]	PharmTargetDB, ChEMBL	Free	Web server	2017	http://lilab.ecust.edu.cn/pharmmapper/

Abbreviations: ACID, auto in silico consensus inverse docking; AFDB, AlphaFold Protein Structure Database; BindingDB, Bindind Database; ChEMBL, a large‐scale database of bioactive molecules with drug‐like properties; COBRA, Collection of Bioactive Reference Analogs; CTD, Comparative Toxicogenomics Database; DNP, Dictionary of Natural Products; DRAR‐CPI, a tool for predicting Drug Repositioning potential and adverse drug reaction (ADR) via CPI; GO, Gene Ontology; KEGG, Kyoto Encyclopedia of Genes and Genomes; MDDR, MDL drug data report; MolTarPred, a resource for molecular target prediction; PDB, Protein Data Bank; PDTD, potential drug target database; SEA, similarity ensemble approach; TarFisDock, Target Fishing Dock; TarPred, a web tool for predicting therapeutic and side effect targets of chemical compounds; UniProt, Universal Protein Resource; ZINC, a Ultralarge‐Scale Chemical Database.

#### 4.1.1 Ligand‐Based 2D Similarity Searching

*TarPred*. TarPred is a computational tool designed to predict targets associated with drug efficacy and adverse effect. It is based on a k‐nearest‐neighbor algorithm (KNN) algorithm using two‐dimensional (2D) ligand similarity searching and data fusion strategies [112, 129]. TarPred integrates information on 533 targets and 179,807 active ligands. Users can input compound structures in interactive graphical or SMILES format, after which TarPred outputs the top 30 predicted interaction targets. These results can be used for drug target prediction and prioritization. In addition, TarPred provides disease information associated with the predicted targets, thereby assisting users in evaluating the biological relevance of predicted results [112]. The predictive performance of TarPred has been validated using Entecavir, a known target‐directed drug used for chronic hepatitis B virus infection [130].

*TargetHunter*. TargetHunter is a target prediction tool based on the 2D structural similarity of small molecules. After users input a compound structure, TargetHunter generates the corresponding molecular fingerprint and compares it with compounds in chemical genomics database ChEMBL. TargetHunter uses the Targets Associated with its MOst SImilar Counterparts algorithm to predict potential biological targets and off‐targets. TargetHunter then ranks the top candidate targets according to similarity scores [113]. Notably, TargetHunter also integrates BioassayGeoMap, which allows users to search for potential collaborators in related research areas. This function may facilitate cross‐team collaboration and promote target discovery and drug repositioning studies involving NPs.

*MolTarPred*. MolTarPred is a web‐based tool for molecular target prediction [114]. Users input the structure of a compound of interest, and MolTarPred returns predicted target results within approximately 1 min. To facilitate interpretation and comparison, the output displays both the query molecule and structurally similar compounds associated with the predicted target [114]. A reliability score is also provided to improve the interpretability and confidence of prediction results. MolTarPred has shown potential utility in target identification for anticancer drugs, orphan drugs for rheumatoid arthritis, and drug repurposing studies [131, 132, 133].

#### 4.1.2 Ligand‐Based 3D Similarity Searching

*ChemMapper*. ChemMapper predicts potential targets based on three‐dimensional (3D) molecular similarity. Its database contains more than 350,000 bioactive compounds with target annotations and over 3 million unannotated compounds [115]. ChemMapper provides two computational modes: Target Navigator and Hit Explorer, both of which support target inference. Users input a compound of interest, and ChemMapper automatically convert its 2D structure into a 3D structure. The platform employs the SHAFTS method, which combines molecular shape overlay and pharmacophore feature matching, to perform 3D similarity searching, ranking, and structural alignment. The most similar compounds and their annotated information are then returned. Based on these structurally similar compounds, ChemMapper infers compound–protein networks and predicts potential protein targets [115]. ChemMapper has been widely applied in virtual screening, chemical genomics, drug repurposing, polypharmacology, and target discovery [134, 135].

#### 4.1.3 Multidimensional Structural Similarity Comparison

*SwissTargetPrediction*. SwissTargetPrediction predicts small‐molecule targets based on both 2D and 3D similarity to known ligands [116]. In 2019, the platform was updated by incorporating a larger amount of interaction data from the ChEMBL database. The updated dataset includes 376,342 compounds, representing a 34% increase, as well as 3068 protein targets and 580,496 binding activities records. This expansion improves the platform's ability to predict additional target proteins [117].

#### 4.1.4 Statistics‐Based Ligand Similarity Assessment

*Similarity Ensemble Approach*. The similarity ensemble approach (SEA) is a statistics‐based chemoinformatics method that evaluates ligand similarity across target‐associated compound sets to predict new targets and off‐targets [111]. The SEA database integrates compound and target information from resources such as ChEMBL and MDL drug data report (MDDR). It uses daylight molecular fingerprints to calculate compound similarity and clusters similar compounds according to their known targets. By simply inputting the SMILES code of a small molecule, users can obtain potential target predictions [118].

#### 4.1.5 Ligand Similarity Searching and Molecular Docking‐Based Approach

*LigTmap*. LigTmap is an online tool for small‐molecule target identification and activity prediction based on ligand‐ and structure‐informed strategies. It provides a fully automated target prediction workflow and returns ligand docking structures in PDB format [119]. Experimental validation showed that the success rate for the top 10 predicted targets generated by LigTMap is approximately 70%, suggesting that its performance is comparable to that of SwissTargetPrediction and SEA [119].

#### 4.1.6 Topological Pharmacophore Features‐Based Similarity Comparison

*Fragment‐Based Method*. Fragment‐based pharmacophore comparison algorithms operate independently of global chemical structure similarity and 3D target structures, making it particularly suitable for structurally complex NPs. This algorithm decomposes NPs into pharmacophore fragments and compares these fragments with those of control drugs with known targets, thereby inferring potential pharmacological targets. Using this strategy, potential macromolecular targets for NP‐derived fragments were identified and applied to the macrolide anticancer drug archazolid A, revealing novel targets that were not predict by other approaches. Its accuracy has been experimentally validated, supporting its potential application in efficient target prediction for NPs [120, 121].

### 4.2 Receptor‐Based Target Prediction

Since the development of the first molecular docking software, DOCK [136], molecular docking has evolved into a mature technology. Generally, molecular docking requires at least two structural inputs: the 3D structure of the target protein and the 2D or 3D chemical structure of the compound. In most cases, users also need to define the binding sites of interest based on prior structural or functional knowledge, thereby enabling simulating of ligand binding within the receptor pocket and improving docking accuracy [137]. Structure‐based docking methods can be widely applied in ligand optimization [138], target–ligand interaction analysis, and virtual screening. In addition, when combined with large‐scale protein structure datasets, molecular docking can be used to identify unknown target proteins for known compounds. This strategy is generally referred to as reverse docking [137] (Figure 6B).

#### 4.2.1 Target Fishing Dock

Target Fishing Dock, also known as TarFisDock, was developed in 2006 as an online web server for target prediction based on reverse docking. Users are required to input small‐molecule structures in mol2 format. TarFisDock automatic searches for potential small molecule‐protein interactions across a large number of protein structures to predict candidate proteins [122]. In TarFisDock, the built‐in potential drug target database (PDTD) is combined with a reverse docking program. The workflow first retrieves proteins from the database that may bind to the input molecule, followed by ligand–protein reverse docking strategy to evaluate potential interactions with these candidate proteins.

#### 4.2.2 DRAR‐CPI

DRAR‐CPI is a web server designed to identify drug repositioning potential and adverse drug reactions (ADRs) by evaluating compound–protein interactions (CPI), and it can also be applied for target discovery. Users input mol2 files of drug molecules with pre‐added charges and hydrogens. DRAR‐CPI checks the file format and uses the DOCK algorithm to calculate the interaction profile between the compound and potential targets [123]. The platform outputs drugs with similar or opposite interaction profiles to the query molecule, suggesting potential new indications, adverse reactions, and candidate off‐targets interacting with the input compound.

#### 4.2.3 IdTarget

IdTarget is a web server that predicts potential small‐molecule targets using a divide‐and‐conquer docking strategy and the AutoDock4 scoring function [139, 140]. After users input ligand files, idTarget performs computational screening and outputs a ranked list of candidate targets after several hours of calculation [124]. In one study, quercetin was subjected to reverse screening using SHAFTS and idTarget, leading to the prediction of multiple protein targets. Based on these results, the potential relevance of the predicted targets to the effects of quercetin in neurodegenerative diseases, cancer, and other diseases was further discussed [141].

#### 4.2.4 GalaxySagittarius

Computational cost of molecular docking is relatively high, making it impractical for exhaustive screening against entire protein structure databases. GalaxySagittarius addresses this issue by integrating docking algorithms with ligand similarity and receptor structure‐based methods for target prediction. By using a protein target database capable of evaluating ligand binding, GalaxySagittarius can identify protein targets with unknown interactions [142]. In a network pharmacology analysis of spermidine for osteoarthritis treatment, GalaxySagittarius was used for target prediction. Cross‐validation identified aryl hydrocarbon receptor as a potential target, and subsequent in vitro and in vivo experiments confirmed that spermidine exerts anti‐inflammatory effects through interacting with this target [143]. An updated version, GalaxySagittarius‐AF, incorporate AlphaFold‐predicted protein structures together with corresponding binding sites and ligands. This new version translates protein structure prediction results into compound target predictions, substantially expanding target coverage and increasing computational speed by approximately 2–3 folds [125].

#### 4.2.5 Auto in Silico Consensus Inverse Docking

Auto in silico consensus inverse docking (ACID) is a web‐based computational tool developed through systematic evaluation of multiple inverse docking methods for target prediction. It integrates automated workflows with consensus inverse docking strategy [126], which yields higher prediction success rates than single docking algorithm. In ACID, a subset of 195 high‐quality complex structures from the PDBbind database was selected to evaluate docking and scoring performance. In addition, 831 target entries and 2086 compound entries were collected from multiple databases. A test set of 51 representative drugs were used to compare the performance of ACID with that of other docking algorithms. ACID has also been applied in NP target discovery and drug repurposing. Notably, it can identify not only on‐target proteins but also potential off‐targets [126].

#### 4.2.6 PharmMapper

PharmMapper is a web‐based tool that predicts small‐molecule targets using reverse pharmacophore matching [127]. It predicts compound targets by matching input small‐molecules against a built‐in pharmacophore model database and ranks predicted targets according to normalized fit scores. The updated version of PharmMapper includes improved methods for identifying pharmacophore features of targets. Its built‐in database, PharmTargetDB, integrates target annotations from BindingDB, TargetBank, DrugBank, and PDTD databases. PharmTargetDB covers 23,236 proteins, including 16,159 druggable pharmacophore models and 51,431 ligandable pharmacophore models, with target information spanning 450 indications and 4800 molecular functions [128]. Overall, PharmMapper captures potential target‐binding patterns by using the 3D interaction features of receptor‐binding sites, making it suitable for candidate target discovery and subsequent experimental validation screening in NP research.

### 4.3 Network Pharmacology and Systems Biology

Network pharmacology and systems biology‐based methods contribute to NP target discovery not primarily by identifying a single definitive hit target, but by organizing multidimensional perturbation profiles induced by NPs into computable networks or feature spaces. These approaches enable candidate prioritization and mechanistic interpretation through the integration of multiple lines of evidence [144]. Unlike methods relying solely on structural similarity or individual docking score, network‐based approaches emphasize multisource evidence integration and context‐dependent interpretation. Therefore, they are particularly suited for addressing the polypharmacological effects and context‐dependent activities commonly observed with NPs (Table 3).

**TABLE 3:** Computational tools based on network pharmacology and systems biology.

Tool name	Data category	License type	Availability status	latest date	Website
Cytoscape [145, 146]	Networks	Free of charge	Web server	2025	https://web.cytoscape.org
GenomeCRISPR [147]	Genomics	Not specified	Web server	2017	http://genomecrispr.org
GT‐Scan [148]	Genomics	Not specified	Web server	2014	https://gt‐scan.csiro.au/
CMap [149, 150, 151]	Genomics	Free	Web server	2017	https://clue.io/l1000‐query
ChEMBL [152, 153]	Compound structures, functions, targets, ADMET	Free for industry and academic users	Web server	2025	https://www.ebi.ac.uk/chembl/
TDR Targets [154, 155]	Genetic, genomic, biochemical, structural, pharmacological data	Open access	Web serve	2021	https://tdrtargets.org
OTTER [156]	Omics, phenotypes	Free	Web server	2023	http://otter‐simm.com/otter.html

Abbreviations: ADMET, absorption, distribution, metabolism, excretion and toxicity; CMap, Connectivity Map; GenomeCRISPR, a database for high‐throughput clustered regularly interspaced short palindromic repeats associated protein 9 (CRISPR–Cas9) screening experiments; GT‐Scan, Genome Target Scan; OTTER, Omics and Text‐based Target Enrichment and Ranking; TDR Targets, a database for tropical disease pathogens.

#### 4.3.1 Cytoscape

Cytoscape is a versatile open‐source platform for visualizing and analyzing large‐scale biomolecular interaction networks [145]. Experimental data, such as expression ratios or protein abundance changes, can be assigned as attributes to nodes or edges and further annotated using established knowledge bases [145]. Cytoscape provides multiple network layout algorithms and filtering tools to optimize network visualization and focus on candidate key nodes [145]. In addition, Cytoscape supports functional expansion through user‐installed plugins, making it adaptable to diverse network pharmacology and systems biology workflows [144, 145]. Although Cytoscape is highly useful for interpreting relationships between functional genomics, proteomics, and biological networks, it does not itself predict all possible interactions between pathogenic factors and regulatory genes [144]. Cytoscape.js provides a JavaScript library for interactive visualization, analysis, and rendering of biological networks in web browsers [157, 158]. The latest Cytoscape Web further establishes an online platform that preserves the interface and key visualization functionality of previous versions while supporting integration with web databases [146].

#### 4.3.2 GenomeCRISPR

Systematic genome‐wide functional screening plays an important role in elucidating gene functions in biological processes. Advances in high‐throughput technologies have provided new strategies for rapid genome annotation and functional interpretation [159]. CRISPR/Cas9 is a powerful gene‐editing technology. Based on CRISPR/Cas9 high‐throughput screening data, the GenomeCRISPR database was developed and contains more than 550,000 single‐guide RNA records from CRISPR high‐throughput screening across 48 human cell lines [147]. The information available in the GenomeCRISPR can support the identification of NP‐related targets by enabling systematic screening, prioritization, and validation of genes functionally associated with drug responses. It is therefore useful for clarifying the MOA of NPs and identifying candidate therapeutic targets.

#### 4.3.3 Genome Target Scan

Genome Target Scan (GT‐Scan) is a web‐based tool that ranks potential targets according to the number of off‐targets within a user‐specified genomic range [148]. GT‐Scan allows users to customize simple target selection rules, such as target length and target site constraints. Several computational tools have been developed for CRISPR/Cas9 guide RNA design and off‐target prediction. For instance, some tools facilitate sgRNA selection and off‐target assessment [160], while Cas‐OFFinder identifies potential off‐target sites for RNA‐guided engineered nucleases [161]. However, these tools do not always support user‐defined rules. CasOT enables genome‐wide Cas9/gRNA off‐target searching, but it is implemented as a Perl script and cannot be used directly online [162]. By contrast, GT‐Scan provides an online and customizable alternative for target selection. In addition, it may also be useful for identifying potential targets in systems beyond CRISPR/Cas‐based applications.

#### 4.3.4 Connectivity Map

Connectivity Map (CMap) is a gene expression profile database designed to reveal relationships among drugs, genes, and diseases through pattern matching and comparison of transcriptomic signatures [149, 150]. It uses connectivity scores based on Gene Set Enrichment Analysis (GSEA) to measure similarity between perturbation‐induced gene expression profiles. In 2017, the L1000 platform, a low‐cost and high‐throughput reduced representation reduced gene expression method, was developed to update CMap. This advancement enabled the generation of large‐scale small‐molecule perturbation datasets and gene expression profiles, facilitating off‐target identification, correct target enrichment, and deeper exploration of the biological functions linking among drug mechanisms, genomes, and diseases [151]. Building upon the CMap framework, a deep learning (DL)‐based efficacy prediction system was developed. This system uses GSEA to assess therapeutic efficacy by taking disease‐associated gene expression changes as input [163].

#### 4.3.5 ChEMBL

ChEMBL is a chemical genomics database originally developed in 2012 [152]. It primarily contains information on the biological activities of molecules and their associated biological targets, integrating data on compound structures, biological functions, targets, and ADMET properties, including absorption, distribution, metabolism, excretion, and toxicity. In recent years, ChEMBL has undergone multiple updates and has become one of the most widely used databases in drug discovery and pharmaceutical chemistry [164]. Several databases, such as PubChem BioAssay, support cross‐access to ChEMBL [165]. The latest version was released in 2024 [153].

#### 4.3.6 TDR Targets

The TDR Targets database was developed in 2008 to facilitate drug development, rapidly identifying molecular targets, and support target prioritization, especially for neglected human diseases pathogens. TDR Targets integrates genetic, genomic, biochemical, structural, and pharmacological data from multiple sources to identify and prioritize drug–target interactions (DTIs), thereby promoting drug repurposing research targeting pathogen‐related molecules [166]. Since its establishment, TDR Targets has been updated multiple times, has evolved to TDR6, namely, version v6.1 [154, 155]. TDR6 contains information on approximately 2 million bioactive compounds and provides three major functions, including network‐driven genome‐wide target prioritization, drug repurposing exploration, and candidate target identification for orphan compounds [155].

#### 4.3.7 Omics and Text‐Based Target Enrichment and Ranking

Omics and Text‐based Target Enrichment and Ranking (OTTER) is a target prediction tool based on omics information and knowledge mining [156]. Unlike many ligand‐based prediction methods, OTTER does not require chemical structure comparison of NPs. Instead, users only need to provide a keyword of interest and omics data as input. OTTER can then rapidly perform target enrichment for small molecules associated with specific phenotypes through text mining. OTTER predicts targets through a three‐step process. First, it performs text mining in all PubMed abstracts using the user‐provided keyword and generates a text score for each differentially expressed gene (DEG). Second, it mines PPIs associated with DEGs that have higher text score and generates corresponding PPIs scores. Third, OTTER integrates the text and PPIs scores into a normalized total score and outputs visualized prediction results. The top 50 DEGs are ultimately presented as candidate targets [156].

### 4.4 Machine Learning and AI

Machine learning (ML) and AI approaches are advancing NP target identification toward a new paradigm based on data‐driven representation learning and probabilistic ranking. Large‐scale model training enables target probabilities estimation across broad target spaces, thereby improving early‐stage screening efficiency [27, 28]. By leveraging strong data integration and predictive capabilities, AI‐based approaches can form a closed loop with downstream experimental validation. However, their reliability is highly dependent on the coverage, balance, and annotation quality of training datasets. This section introduces several AI‐based strategies applicable to target identification, which can be categorized into four classes based according to their methodological principles (Table 4).

**TABLE 4:** Computational tools based on machine learning and artificial intelligence.

Tool name	Method	License type	Availability status	Latest version	Website
HitPick [167, 168]	2D and 3D structural similarity, ML	Free	Web server	2018	www.hitpickv2.com
SuperPred [169, 170, 171]	2D and 3D structural similarity, ML	Publicly available without registration	Web server	2022	https://prediction.charite.de/index.php
PASS [172, 173]	2D chemical structures, ML	Free registration	Web server	2018	https://www.way2drug.com/passonline/
COMET [174]	ML	Free registration	Web server	2025	http://www.pdbbind‐plus.org/comet
STarFish [175]	ML, model stacking	Free (datasets, source code, and API)	Public Github repository	2019	https://github.com/ntcockroft/STarFish
DisGeNET [176, 177, 178]	NLP	Free academic access	Web server	2025	https://www.disgenet.com
HIT [179, 180]	NLP	Free for academic	Web server	2022	http://hit2.badd‐cao.net
FRoGS [181]	DL	Not specified	Public Github repository	2024	https://github.com/chenhcs/FRoGS
DeepPurpose [182]	DL	Not specified	Public Github repository	2024	https://github.com/kexinhuang12345/DeepPurpose
MINN‐DTI [183]	DL	Not specified	Public Github repository	2022	https://github.com/admislf/MINN‐DTI
DLM‐DTI [184]	DL, dual language model	Not specified	Public Github repository	2024	https://github.com/jonghyunlee1993/DLM‐DTI_hint‐based‐learning/tree/master
GCN‐DTI [185]	DL	Not specified	Public Github repository	2020	https://github.com/zty2009/GCN‐DNN/

Abbreviations: 2D, two‐dimensional. 3D: three‐dimensional; API, application programing interface; COMET, combined matrix for elucidating targets; DeepPurpose, a deep learning library for drug–target interaction prediction; DisGeNET, a comprehensive platform integrating information on human disease‐associated genes and variants; DL, deep learning; DLM‐DTI, a dual language model‐based DTI model; FRoGS, functional representation of gene signatures; GCN‐DTI, graph convolutional network (GCN)‐DTI; HIT, Herb Ingredients’ Targets; HitPick, a web tool for hit identification and target prediction of chemical screenings; MINN‐DTI, a model for drug–target interaction (DTI) prediction with mutual interaction neural network; ML, machine learning; NLP, natural language processing; PASS, prediction of activity spectra for biologically active substances; STarFish, stacked ensemble target fishing; SuperPred, a web server for predicting anatomical therapeutic chemical (ATC) codes and compound targets.

#### 4.4.1 Structure‐Based ML Model

*HitPick*. HitPick is a web server for identifying hits and predicting human protein targets in high‐throughput chemical screening. It combines 2D one‐nearest‐neighbor similarity searching with Laplacian‐modified Naïve Bayesian target models based on ML. Using bioassay data as input, HitPick can output a hit list and predict corresponding targets. It can also directly predict targets for selected compounds. Its target prediction performance is comparable to that of the SEA method [167]. The advanced version, HitPickV2, can predict up to 10 targets from 2739 human proteins for each compound [168]. It also provides a rank score for each predicted targets together with structure similarity analysis based on of known compounds. Users need to input a list of SMILES strings to query compound 2D structures and potential targets. The target data are derived from the Reactome database [186]. Recent studies have combined HitPick with other computational methods for biological target analysis [187].

*SuperPred*. SuperPred is a web server for predicting anatomical therapeutic chemical (ATC) codes and compound targets using the ATC classification framework recommended by the World Health Organization [169, 170, 188]. By predicting ATC codes or molecular targets, SuperPred provides valuable information that can support drug development. Following its 2022 update, SuperPred has been upgraded into a new server based on ML models. The updated version uses logistic regression and ECFP4 fingerprints and expand the prediction scope from active binders to experimentally validated nonbinders extracted from the ChEMBL database [171]. By integrating physicochemical properties and similarity searching, SuperPred can predict new targets and indications for compounds, and may support drug repurposing and adverse effect prediction.

*Prediction of Activity Spectra for Biologically Active Substances*. Prediction of activity spectra for biologically active substances (PASS) utilizes molecular structural features to predict interactions between compounds and biological targets based on 2D chemical structures. It can predict thousands of biological activities and provides online prediction of biological activity profile [189]. Based on the structural formula of a substance, PASS applies ML algorithms to predict more than 4000 biological activities, including pharmacological effects, MOA, toxicity, adverse reactions, interactions with metabolic enzymes and transport proteins, and effects on gene expression [173]. PASS has been successfully applied to evaluate the biological activities of various NPs, including sponge alkaloids and lupane triterpenoids [172, 190, 191]. However, PASS has limitations in assessing the impact of stereoisomerism on biological activity [173]. Importantly, predicted biological activities and targets still requires experimental validation.

*Combined Matrix for Elucidating Targets*. The core concept of combined matrix for elucidating targets (COMET) integrates ligand‐based similarity scoring and target‐based binding affinity prediction within a unified framework to narrow candidate targets across a large‐scale target space [174]. COMET uses the chemical structure of a query molecule, such as a SMILES string, as input. It first conducts ligand‐driven screening using multiple similarity metrics and strategies, including ECFP4 fingerprints and Tanimoto similarity, to generate an initial candidate set. Subsequently, in the target‐driven module, COMET incorporates the DL scoring algorithm PLANET to predict binding affinity based on target pocket representations. These multisource features are then input into a random forest model to generate probability scores and ranking lists for candidate targets [174]. Optionally, graphic processing unit (GPU)‐accelerated molecular docking can be performed for selected candidates to provide predicted binding poses. However, the overall runtime is largely determined by the docking step. By combining ligand‐based recall and target‐based ranking, COMET balances speed and accuracy and facilitates target prioritization for downstream experimental validation. Nevertheless, its performance depends heavily on the coverage of target–ligand pairs in reference databases and the quality of molecular structures and binding pocket information. Therefore, its application may be limited for targets with sparse ligand annotations or unreliable structural data [174].

*Stacked Ensemble Target Fishing*. Stacked ensemble target fishing (STarFish) is a web application that conducts target fishing for NPs using stacking KNN models [175]. STarFish uses a synthetic dataset containing 107,190 compound–target pairs as the training set and trains stacked classifiers with transformed feature combinations. For benchmark testing, a dataset was constructed by integrating multiple NP databases, covering 5589 compound–target pairs, 1943 compounds, and 1023 targets [175]. The high performance of STarFish in testing indicates its promising potential for NP target discovery.

#### 4.4.2 Knowledge‐Based Natural Language Processing

*DisGeNET*. DisGeNET uses gene‐disease associations (GDAs) and variant‐disease associations (VDAs) as core data units. It systematically integrates multisource data from expert‐curated resources, GWAS catalogs, animal models, and literature information mined via natural language processing (NLP). To address retrieval and integration challenges caused by cross‐database fragmentation and conceptual heterogeneity, DisGeNET performs standardized semantic annotation of diseases, phenotypes, and association types [176]. Users can input single or multiple identifiers for diseases, genes, or variants. The outputs provide GDA or VDA results together with phenotypic and prioritization‐related metrics to support candidate target ranking. Because DisGeNET integrates text‐mined information and heterogeneous database integration, network pharmacology applications should apply stratified filtering and interpretation based on source type, score, and evidence level to reduce potential data bias [176, 177]. The latest release of DisGeNET integrates information on drugs and chemicals, and incorporates environmental factors associated with complex diseases, thereby expanding both the data types and existing platform functionalities [178].

*Herb Ingredients’ Targets*. Herb Ingredients’ Targets (HIT) is a knowledge‐based database linking herbal ingredient to their target proteins. It uses PubMed abstract‐based text mining and manual screening to construct a keyword library, connecting individual TCM ingredients with reported target proteins [179]. In 2022, the database was upgraded and released as HIT 2.0. The updated version incorporated NLP‐based AI technology through PubTator Central, enabling automatic annotation of newly published PubMed abstracts. In addition, target‐mining and user‐curation functions were introduced to support real‐time updating of relevant information [180].

#### 4.4.3 Multiomics‐Based DL Model

*Functional Representation of Gene Signatures*. Multiomics big data can be used to identify gene signatures for biological target prediction. The functional representation of gene signatures (FRoGS) strategy was developed by training a DL model [181]. Using a neural network model that takes FRoGS vector representations as input to compute signature similarity, the method was evaluated using the Broad Institute L1000 datasets. FRoGS outperformed models relying solely on gene identities for compound–target prediction. The AI model used in FRoGS integrates pharmacological activity data with large‐scale omics information from compounds, cell types, disease models, and patient cohorts. This integration improves the success rate of compound–target discovery and demonstrates the potential of functional representation learning for identifying new therapeutic targets.

#### 4.4.4 Interaction‐Based DL Method

Exploring potential interactions between drugs and proteins is an initial and essential step in drug design and development [192]. Accurate prediction of complex and previously unknown DTIs is particularly important for NP target discovery, as DTI mining can facilitate the identification of potential drug targets. However, traditional computational methods, including scoring functions, random forests, and support vector machines, often show limitations in predictive performance [193, 194, 195]. DL models now provide new strategies DTI prediction and typically include three modules: drug feature extraction, target feature extraction, and interaction prediction [183]. Large language models (LLMs), including ChatGPT‐like architectures, have also promoted the development of DTI research and may contribute to DTI prediction [196].

*DeepPurpose*. DeepPurpose is a DL‐based framework for training DTI prediction models using more than 50 neural network architectures, eight compound encoders, and seven protein encoders. Users are required to input pairs of SMILES strings and protein amino acid sequence [182]. DeepPurpose has been compared with other advanced DL methods, and most DeepPurpose models showed comparable predictive performance on two benchmark datasets [197, 198]. However, DeepPurpose is developed based on Pytorch and therefore requires users to have a certain level of programming proficiency.

*MINN‐DTI*. MINN‐DTI is a DTI prediction platform based on mutual interaction neural network (MINN). It improves the interpretability of DTI mechanisms by adjusting the weights assigned to amino acids and atoms [183]. MINN‐DTI integrates interacting‐transformers and improved communicative message passing neural network modules to construct an interaction neural network as the core component of the tool. It also includes a target preprocessing network and an interaction prediction network, providing more comprehensive DTI characterization and improving prediction capability. The core principle of MINN‐DTI is to model interactions between drug molecules and target proteins using multiscale deep neural networks (DNNs). Specifically, drug molecules and target proteins MINN‐DTI are represented as graph structures, and graph neural networks methods are used to extract features. The model was evaluated on human dataset, DUD‐E, and BindingDB, and its performance surpassed several existing algorithms across multiple metrics, demonstrating strong predictive capability.

*DLM‐DTI*. Transformer‐based large models typically require large‐scale datasets to fully realize their predictive potential. By contrast, DLM‐DTI was developed as a dual language model for DTI prediction that can perform effectively in relatively smaller‐scale settings using a prompt‐based learning strategy [184]. DLM‐DTI consists of three components: a drug encoder, based on ChemBERTa [199]; a target encoder; and an interaction prediction head. To construct the target encoder, the model uses ProtBERT and introduces teacher and student language models for protein sequence representation. DLM‐DTI was trained and evaluated on the BIOSNAP [200], DAVIS [197], and BindingDB [201], where it demonstrated predictive advantages. Nevertheless, its ability to predict binding for cold‐start targets and cold‐start drugs still requires further improvement. Although DLM‐DTI is a large model‐based approach, it is computationally efficient and can perform prediction tasks on conventional GPUs.

*Graph Convolutional Network‐DTI*. Graph convolutional network (GCN)‐DTI was designed to improve DTI discrimination by modeling relational dependencies among drug–protein pairs (DPPs) [185]. Its prediction workflow consists of three steps. First, a weighted DPP network is constructed, in which each candidate drug–protein pair is represented as a graph node. Second, feature information is extracted using a GCN. An initial vector is constructed for each DPP node, and graph convolution is used to aggregate neighborhood information on a weighted graph to obtain representations incorporating topological constraints. Third, a DNN classifier is trained as a supervised learning model. Using GCN‐derived embeddings, it outputs the probability that a given DPP represents a true interaction, which is then used to rank candidate targets [185]. GCN‐DTI integrates drug networks and protein networks within a unified graph learning framework, helping to reduce false‐positive predictions. However, the large scale of DPP nodes imposes substantial computational and storage burdens, which remains the major bottleneck of this approach [185].

## 5 Integrated Experimental–Computational Discovery Pipelines

AI has become increasingly integrated into real‐world biomedical applications and is playing an expanding role in drug discovery [191]. Advances in computer‐aided drug design and AI drug discovery have accelerated target discovery process for NPs [202]. In the preceding sections, we separately summarized major experimental and computational technologies used for NP target identification. However, as the polypharmacology and weak‐affinity binding characteristics of NPs become increasingly recognized [17], traditional linear strategies are more likely to accumulate false‐positive findings or overlook critical targets within complex biological systems [46]. Therefore, NP target discovery should move beyond the use of isolated methods and toward reusable, integrated experimental–computational workflow. By combining computational prediction, experimental screening, orthogonal validation, and representative case‐based reasoning, such workflows can improve the robustness, reproducibility, and translational potential of target attribution [26, 27, 79].

### 5.1 Iterative and Closed‐Loop Discovery Strategies

Traditional NP target discovery has often followed a linear research paradigm, starting with target prediction, proceeding to target validation, and ending with a mechanistic conclusion [46, 79]. However, as the intrinsic complexity of NPs have become increasingly evident [16, 17], unidirectional workflows have shown clear limitations in practical applications. These workflows may lead to the accumulation of false‐positives candidates or the omission of genuine functionally relevant targets [18, 46]. In recent years, iterative and closed‐loop strategies integrating AI‐based prediction with experimental validation have emerged as more robust research paradigms [26].

In an iterative discovery strategy, computational biology tools or large‐scale AI models are first used to generate an initial set of target hypotheses [118, 203]. These hypotheses are then evaluated through phenotypic assays, chemical proteomics, thermal stability analysis, or other experimental approaches. Experimentally supported hits are subsequently fed back into the computational framework to refine, constrain, and re‐rank the candidate range space, thereby initiating the next round of prediction and screening [203]. Through repeated cycles of computational prioritization and experimental feedback, target confidence can be progressively improved. This strategy is particularly suitable for NP research, where multitarget regulation, weak binding affinity, and context‐dependent activity are common.

**FIGURE 7:**
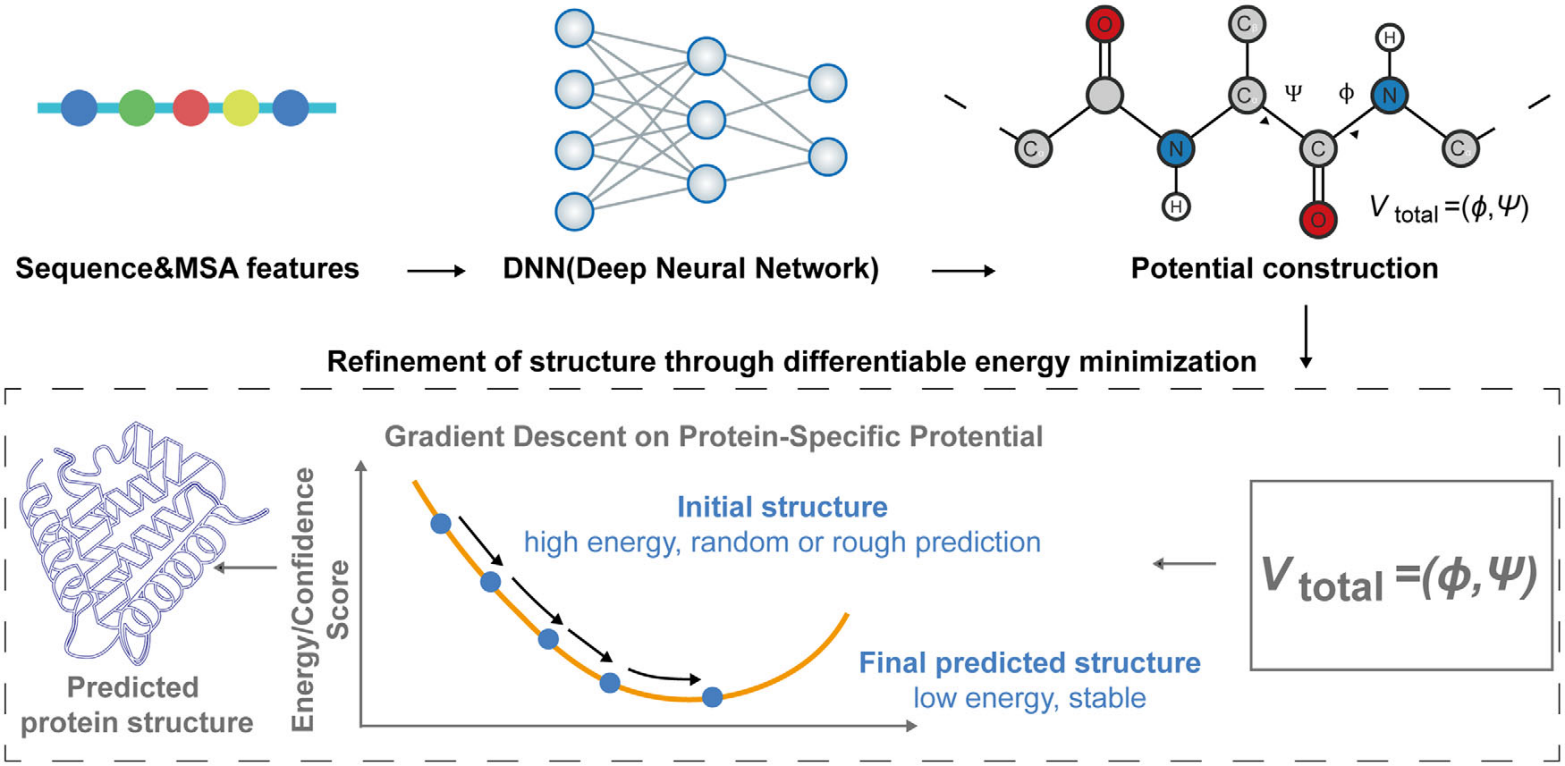
Structure‐driven target discovery workflows in the AlphaFold era. Deep learning‐based protein structure prediction pipelines leverage protein sequences and multiple sequence alignment (MSA) features to construct protein‐specific representations using neural networks. Differentiable energy is then applied to refine initial high‐energy conformations into stable predicted protein structures. High‐quality predicted structures substantially expand structural accessibility of potential targets and facilitate reverse docking, molecular dynamics simulations, and binding site inference.

Building on this iterative logic, closed‐loop discovery strategies further emphasize the deep integration between computational modeling and experimental validation. Algorithms rapidly narrow the candidate target pool, whereas experimental feedback continuously improves model performance through cross‐validation and iterative optimization [28]. Advances in AlphaFold‐based protein structure prediction have increased the accessibility of structural information for putative targets [204], enabling reverse docking, molecular dynamics simulations, and drug–target binding‐site inference to be incorporated more effectively into closed‐loop workflows (Figure 7). These approaches can be synergistically integrated with omics profiling, cell–cell communication analysis, and phenotype‐ or mechanism‐oriented validation strategies [205, 206]. In parallel, knowledge‐driven models that integrate literature, omics, and network information can support priority ranking of candidate targets, thereby focusing the validation process on more biologically plausible and therapeutically relevant candidates [156, 207].

Overall, iterative and closed‐loop strategies represent a paradigm shift in NP target discovery. They promote more efficient, reliable, and self‐correcting research processes and may help bridge the gap between target prediction, mechanistic validation, and translational application [191].

### 5.2 AI‐Guided Experimental Validation

Although computational algorithms may achieve high theoretical prediction accuracy, target screening and target identification ultimately depend on experimental validation to establish target authenticity and biological relevance. With the widespread application of AI prediction models in target screening, a new validation strategy has gradually emerged in which computational prioritization guides experimental decision‐making. The central objective of this strategy is to reduce experimental burden while preserving mechanistic rigor [26].

AI‐guided experimental validation does not rely on a single prediction output. Instead, ML or DL‐based target prediction tools, such as GalaxySagittarius‐AF and STarFish, integrate multidimensional information to stratify and rank candidate targets. This prioritization directs experimental validation toward target subsets with stronger biological plausibility or disease relevance, thereby improving the overall efficiency of target discovery. Under AI guidance, experimental validation typically proceeds from indirect to direct evidence. Initial validation is often performed at the phenotypic level using cellular or animal models to determine whether predicted targets are associated with observed pharmacological effects. For early‐stage target engagement screening, label‐free or stability‐based approaches, including DARTS and CETSA, are commonly applied to evaluate drug–protein interactions target engagement that approximate physiological environments [40, 76]. After stability‐based screening, candidate targets can be further examined using direct chemical proteomics, such as ABPP and CCCP, as well as functional perturbation studies. These experiments provide evidence of binding, regulation, and biological causality, thereby supporting mechanistic interpretation and mechanism‐of‐action analysis [14, 46, 208].

It needs to be emphasized that AI guidance is not intended to replace experimental validation or diminish its central role in target discovery. Rather, it serves as an auxiliary decision‐support strategy. In complex biological systems, computational models can guide the optimization of experimental design, reduce redundant assays, and accelerate the identification of potential therapeutic targets [26].

### 5.3 Representative Workflows and Best Practices

#### 5.3.1 Workflow A: Algorithm‐Prediction‐Driven Candidate Target Identification

In algorithm‐prediction‐driven workflows, input information typically falls into three categories: (i) molecular fingerprints or molecular descriptors representing chemical structure [168, 182]; (ii) target‐related information such as protein amino acid sequences or structural features [182]; and (iii) drug‐induced omics signatures [156]. Multiple target prediction tools can then be applied with predefined decision rules, such as intersection analysis, weighted scoring, or integrated prioritization, to generate a refined list of candidate targets for experimental validation [113, 156, 168, 187].

A representative example is the target identification study of sanguinarine in oral squamous cell carcinoma. In this study, researchers first used a proteome‐level approach limited proteolysis–MS to identify six candidate proteins. They then applied the GalaxySagittarius algorithm to predict and shortlist the top 10 candidate targets. By integrating these two analytical modes, the study focused on pyruvate kinase M2 (PKM2) as a high‐priority candidate. Importantly, the workflow did not terminate at computational outputs. Instead, direct interaction between sanguinarine and PKM2 was further verified using orthogonal biophysical assays, including surface plasmon resonance (SPR) and microscale thermophoresis (MST). This strategy ultimately confirmed PKM2 as a direct target of Sanguinarine and established a closed‐loop target discovery conclusion [209]. Molecular docking and molecular dynamics simulations are frequently used for preliminary DTI screening and hypothesis generation. However, they are insufficient as standalone evidence of direct binding. Therefore, after computational prediction, at least one orthogonal experiment capable of demonstrating direct target engagement, such as SPR, MST, CETSA, or DARTS, should be included to substantiate target identification [82].

#### 5.3.2 Workflow B: Target‐Binding Evidence and Mechanistic Closed Loop

After an initial list of candidate targets is generated, biochemical approaches such as DARTS can be applied in cell lysates to evaluate target binding [40]. CETSA and TPP can then be used in intact cells or other near‐native systems to identify proteins that undergo compound‐induced stability shifts, thereby capturing high‐confidence candidate targets with interaction‐relevant signatures under physiologically relevant conditions [41, 76]. Based on these data, mechanism‐of‐action hypotheses can be developed and an evidentiary closed loop can be pursued to confirm whether candidate targets make causal contribution to the observed phenotype. For example, aloperine was identified as an autophagy inhibitor in non‐small cell lung cancer cells. DARTS‐based prioritization nominated vacuolar protein sorting‐associated protein (VPS4A) as its direct target. Subsequent immunoblotting, CETSA, SPR, and docking analyses supported binding and helped map key binding determinants. Through genetic perturbation, site‐directed mutagenesis, and rescue experiments following VPS4A knockout, the study showed that aloperine‐induced stabilization of VPS4A was markedly attenuated, thereby validating the functional relevance of binding [210]. Although CETSA and TPP are highly useful for detecting target engagement and generating direct or indirect clues about DTIs, definitive confirmation of direct binding and causal mechanism still requires orthogonal biophysical assays and functional validation. Gene knockout, knockdown, rescue experiments, and site‐specific mutation are particularly important for demonstrating that target engagement is mechanistically responsible for the observed biological phenotype [77].

#### 5.3.3 Workflow C: Chemoproteomics‐Based Validation and Mechanistic Elucidation

For NPs characterized by weak affinity, transient interactions, or covalent reactivity, chemoproteomics‐based approaches are NPs advantageous. Compared with ABPP, CCCP is generally considered a more target‐class‐unbiased strategy because it can identify protein targets even in the absence of enzymatic activity. However, CCCP may generate false‐positive hits and cannot resolve protein activation states, which may compromise interpretability and accuracy. In NP studies, robust target prioritization can be achieved by integrating ABPP with quantitative proteomics workflows, such as stable isotope labeling by amino acids in cell culture (SILAC), tandem mass tag‐based proteomics, or data‐independent acquisition. Once candidate targets are ranked, this quantitative foundation can support further site mapping and mechanism‐of‐action characterization using complementary strategies, thereby enabling deeper mechanistic insights [14, 43]. A representative example involves bavachinin (BVC), an active component of the TCM Psoralea corylifolia, in the context of nonalcoholic fatty liver disease. Researchers synthesized a BVC‐derived probe XQ, and applied click chemistry‐enabled ABPP combined with LC–MS/MS to identify proliferating cell nuclear antigen as a direct target of BVC. During target confirmation, orthogonal evidence from CETSA, DARTS, and SPR further supported direct binding, thereby enabling elucidation of the hepatoprotective mechanism of BVC [211].

From a methodological standpoint, the credibility of chemoproteomics approaches largely depends on rigorous control design. Specificity is commonly inferred through competition with the parent compound, while hits are prioritized according to quantitative enrichment, dose responsiveness, and reproducibility. Orthogonal validation should then be incorporated to reduce false positives arising from any single experimental platform. Before interpreting enrichment results, it is essential to demonstrate that the probe retains the key biological activity of the parent compound and exhibits comparable target‐binding behavior; otherwise, enrichment signals may primarily reflect probe‐specific chemistry rather than the true mechanism of action of the parent NP [18].

## 6 Translational Applications and Representative Case Studies

Building on the integrated pipelines described above, this section illustrates how representative case studies in NP target discovery can be organized within a closed‐loop framework to support translational implementation. It also summarizes key lessons learned from practical applications, thereby providing a more actionable basis for subsequent discussions on precision medicine and drug development [26].

### 6.1 Successful Examples of NP Target Identification

#### 6.1.1 Acevaltrate Induces Ferroptosis in Colorectal Cancer via Dual Target Identification of PCBP1/2 and GPX4

Acevaltrate (ACE), a NP derived from Valeriana jatamansi, inhibits colorectal cancer cell proliferation and induces ferroptosis at the cellular level [212]. By integrating proteomics clues, the study focused validation on the iron chaperone‐related proteins poly(rC)‐binding protein 1 and 2 (PCBP1/PCBP2). Western blotting, DARTS screening, and cellular knockdown experiments were used to evaluate the functional relevance of these candidates. Subsequent CETSA analysis showed that ACE treatment reduced the thermal stability of PCBP1/2, while SPR assay confirmed direct binding between ACE and PCBP1. Further evidence from DTT competition assays, molecular docking based on AlphaFold‐predicted structures, cysteine residue mutagenesis, and MST assays collectively demonstrated that ACE directly binds to PCBP1/2. Mechanistic studies further revealed that ACE promotes GPX4 ubiquitination, linking GPX4 depletion to the ubiquitin‐proteasome pathway. In vivo experiments using a mouse tumor model further supported the therapeutic relevance of PCBP1/2 [212]. Overall, this study established a dual‐mechanism framework in which ACE induces ferroptosis in colorectal cancer through direct targeting of PCBP1/2 and regulation of GPX4 stability.

#### 6.1.2 Homoharringtonine Directly Binds EWSR1 to Suppress Acute Myeloid Leukemia Through m6A–YTHDF2‐Dependent Regulation

To identify the therapeutic target of homoharringtonine (HHT) in acute myeloid leukemia, researchers first synthesized a biotin‐labeled probe, bio‐HHT, and conducted high‐throughput binding screening using the HuProt human proteome microarray [213]. This screening identified 42 high‐confidence candidate proteins. Subsequent pull‐down experiments combined with SILAC‐based quantitative proteomics in leukemia cells revealed significant enrichment of EWS RNA‐binding protein 1 (EWSR1). After EWSR1 was identified as a key target, competitive pull‐down, DARTS, and CETSA provided evidence of intracellular interactions and compound‐induced stability changes. Biophysical techniques, including MST and SPR, further confirmed direct binding between HHT and EWSR1. Domain mapping localized the binding region to the RNA recognition motif (RRM) of EWSR1 [213].

The study then designed a photoaffinity probe, HHT‐PAL, and used LC–MS/MS to identify modified peptides within the RRM. Point mutations experiments, MST, and pull‐down assays further validated the binding affinity of key residues. Finally, transcriptomic sequencing showed a strong correlation between EWSR1 knockdown and HHT treatment. Moreover, EWSR1 knockdown markedly attenuated the antileukemic efficacy of HHT. This convergence of intracellular target engagement, direct binding evidence, and function‐dependent pharmacologic effects strongly supports EWSR1 as a direct target of HHT. It also provides a mechanistic basis for attributing the drug's efficacy to EWSR1‐mediated regulation of YTHDF2–m6A‐associated RNA homeostasis through phase separation [213].

#### 6.1.3 Caffeic Acid Covalently Targets Annexin A5 to Suppress SASP Signaling and Ameliorate Lung Fibrosis

This study first established a quantifiable phenotypic readout based on the reduction of senescence‐associated secretory phenotype (SASP)‐related inflammatory factors, including TNF‐α, IL‐6, IL‐1β, in an aged lung epithelial cell model [214]. Screening a library of NPs and dietary compounds identified caffeic acid (CA) as the most potent SASP inhibitor for further investigation. Based on its chemical structure, CA was considered capable of covalently binding to cysteine residues. Therefore, candidate covalent targets of CA in senescent cells were screened by combining cysteine‐reactive ABPP with competitive chemical proteomics. This strategy identified Annexin A5 (ANXA5) as a potential covalent target. For target validation and site identification, the study demonstrated direct interaction between CA and ANXA5 using in vitro competitive labeling, MST assays, and CETSA. By synthesizing a CA‐derived probe and performing point mutation experiments, the covalent binding site was precisely mapped to Cys316 of ANXA5, advancing the initial target hit to a site‐specific direct binding event [214].

Further mechanistic studies revealed that the covalent interaction between CA and ANXA5 induces ANXA5 protein degradation, thereby inhibiting the PKCθ‐NF‐κB signaling cascade in senescent cells. In addition, siRNA‐mediated knockdown of ANXA5 attenuated the regulatory effects of CA on downstream signaling and inflammatory secretion. These results established a coherent evidence chain linking target engagement, proteostasis alteration, pathway modulation, and phenotypic improvement. Finally, in a mouse model of lung fibrosis, oral administration of CA reduced inflammatory factor levels, improved lung histopathology, and attenuated fibrosis markers. These in vivo effects were consistent with the cellular observations and suppressed signaling pathways, supporting CA as a senomorphic candidate with a defined protein target for anti‐inflammatory and antifibrotic applications [214].

### 6.2 Lessons Learned From Translational Successes and Failures

A major translational bottleneck in NP target discovery lies in progressing from initial associations to reproducible binding evidence and ultimately to functional causal support. Accumulating evidence indicates that sequentially organizing screening‐ or enrichment‐derived leads with orthogonal validation and functionally closed‐loop verification can enhance the robustness and translational potential of target attribution. For instance, CCCP‐related studies often use enrichment strategies and MS to enable rapid and relatively unbiased prioritization of key targets, followed by mechanism‐guided refinement. In brain tissue from a pilocarpine‐induced epilepsy model, affinity chromatography and MS were used to identify proteins that specifically bind curcumin, and Western blot analysis further confirmed protein tyrosine phosphatase receptor type Z1 as a direct target of curcumin, thereby elucidating a microglia‐associated mechanism of action [215]. In non‐small cell lung cancer cells, preparation of the molecular probe WP and quantitative chemoproteomics integrating SILAC with ABPP identified peroxiredoxin 6 as a direct target of Withangulatin A [51]. Similarly, ABPP combined with LC–MS/MS enabled the identification of multiple proteins directly targeted in lipopolysaccharide‐activated macrophages; follow‐up validation showed that Celastrol suppresses sepsis‐associated inflammation and the Warburg effect by targeting PKM2 and HMGB1 [81].

Stability‐oriented label‐free strategies can also support candidate screening in complex biological contexts. DARTS‐MS is commonly used to nominate key targets and, together with downstream validation, to construct translational mechanistic frameworks. Representative examples include DCTPP1 [216], VPS4A [210], PHD2 [217], emphasizing the principle of first establishing reproducible interaction signatures and then iteratively closing the evidence loop at the pathway and phenotype levels. Acetic acid‐treated Artemisia argyi‐derived CDs (AA‐CDs) showed promising therapeutic activity against osteoarthritis. DARTS and CETSA revealed that AA‐CDs target the HSPA5–GPX4 axis to inhibit ferroptosis in chondrocytes [218]. In another study, the dual‐mechanism TME therapeutic strategy, PEOz–FA–anti‐PD1 nanoparticles, was investigated using SPR, DARTS, CETSA, and molecular docking. The results showed that forsythoside A directly binds lysine‐specific demethylase 1 and regulates extracellular vesicles secretion [219]. This nanomedicine strategy significantly enhanced the targeting capability and stability of forsythoside A, offering a novel approach to potentiate anti‐PD1/PD‐L1 therapy [219].

In contrast to studies with robust evidence organization, recurrent controversies in NP research often arise from methodological bias and unclear boundaries in evidence interpretation. Chemical modification and enrichment procedures may constrain applicability, reduce specificity, or alter binding activity. Consequently, control design and probe fidelity frequently determines the credibility of target identification conclusions [34, 39]. Additionally, research on emerging NP‐derived entities still faces numerous challenges. The development of CD‐based targeted therapeutics is constrained by unclear SARs and undefined in vivo safety profiles [12]. Preparation and characterization techniques for NP‐derived nanovesicles remain immature, and multidimensional research approaches are needed to comprehensively elucidate their MOA [220].

Without experimental validation, candidate target screening and mechanistic conclusions based solely on computational prediction tools remain speculative. In studies of curcumin in colorectal cancer and tanshinone IIA in hepatocellular carcinoma, tools such as PharmMapper, together with multidatabase integration, network pharmacology, and molecular docking, generated preliminary mechanistic frameworks. However, experimental validation remains necessary to translate computational leads into actionable conclusions [221, 222]. Moreover, insufficient transparency regarding computational tools, databases, and key workflow parameters can directly compromise reproducibility. For instance, DRAR‐CPI is relatively time consuming for reverse docking and has limited capacity to accurately quantify target specificity [123, 223]. OTTER‐related applications further suggest that literature‐derived knowledge and omics information can enrich and rank candidate targets and guide experimental validation. Nevertheless, limitations such as semi‐automated operation, dependence on single‐keyword input, and hardware requirements may restrict its methodological generalizability [156, 224].

### 6.3 Implications for Precision Medicine and Drug Development

From a precision medicine perspective, integrated multitool prediction combined with network analysis can help identify key genes or proteins from large candidate sets, particularly in complex or multicomponent systems. This provides a foundation for understanding pharmacological mechanisms, expanding therapeutic indications, and designing rational combination therapies [225]. For example, in arthritis models, candidate integration across multiple databases [226, 227], together with functional annotation such as Gene Ontology enrichment analysis [228], can prioritize critical targets. Subsequent experimental validation aligns with the stratified and verifiable features of precision medicine [229]. Within the drug development pipeline, appropriate use of computational tools, in which algorithms drive target identification and experiments provide confirmation, can mitigate the technical barriers and high costs associated with traditional target discovery workflows. When further empowered by AI technologies, such strategies may accelerate the discovery and development of novel NP‐derived therapeutics.

Ultimately, progress toward precision medicine requires operational linkage among target discovery, biomarkers, patient stratification, and clinically quantifiable endpoints. Metabolomics has been widely applied in disease research and can support mechanistic interpretation while providing clues for potential targets [99, 100, 101]. Bile acid metabolomics further incorporates molecular receptor axes, such as farnesoid X receptor‐associated pathways, into pharmacological mechanistic frameworks for acute pancreatitis [230]. In another example, researchers prepared plant‐derived nanovesicles from Portulaca oleracea and used integrated multiomics network analysis, including proteomics and metabolomics, and pathway enrichment, to progressively elucidate their potential mechanisms in diabetic wound repair, thereby supporting novel drug development [231].

The integration of transcriptomic, proteomic, and single‐cell spatial transcriptomic data enables localization of critical cell subpopulations and investigation of pathway enrichment [232], which is important for clinical sample analysis and disease diagnosis. However, limitations in spatial feature recognition, sampling methods, and the high cost of omics sequencing, have hindered the broader application of single‐cell transcriptomics in elucidating the mechanisms of NPs [90]. MS‐based glycoproteomics is an important strategy for glycoprotein analysis [233]. However, the expression abundance of surface‐plasma membrane proteins is typically low. TMEPro, a universal multidimensional clinical functional proteomics strategy, provides a new perspective for enrichment and analysis of the glycosylated surface‐plasma membrane proteome [234]. This approach may also be applicable to other cancer models.

The integration of AI with single‐cell multi‑omics technologies holds promise for improving prediction of NP bioactivity and accelerating candidate target identification [235]. Nevertheless, the scarcity of curated model datasets and the need for reliable large‑scale models remain major challenges [235]. In addition, cross‐omics concepts and methods, including protein‐metabolite interactomics, such as LiP‐SMap and MIDAS, suggest that metabolite signals can be translated into actionable clues localized to specific proteins and binding sites. These approaches provide methodological foundations for integrating NP effects into more refined mechanistic maps and traceable evidence systems [236, 237].

## 7 Current Limitations, Standardization Issues, and Data Gaps

Despite major advances in NP target discovery driven by chemical proteomics and label‐free biophysical methods, target identification for NPs still faces several persistent obstacles. These include insufficient reproducibility, inconsistent data quality and annotation systems, and the absence of unified community standards and shared resources. These issues are closely interconnected. Discrepancies in probe design and experimental systems amplify uncertainties associated with nonspecific binding and weak interactions. Incomplete data annotation and reporting hinder cross‐study integration and computational model training. Moreover, the lack of a unified evidence‐grading system often leads to the conflation of “candidate targets” with “physiologically relevant targets,” ultimately weakening mechanism interpretation and translation potential [14, 238].

### 7.1 Reproducibility and Validation Challenges

**FIGURE 8:**
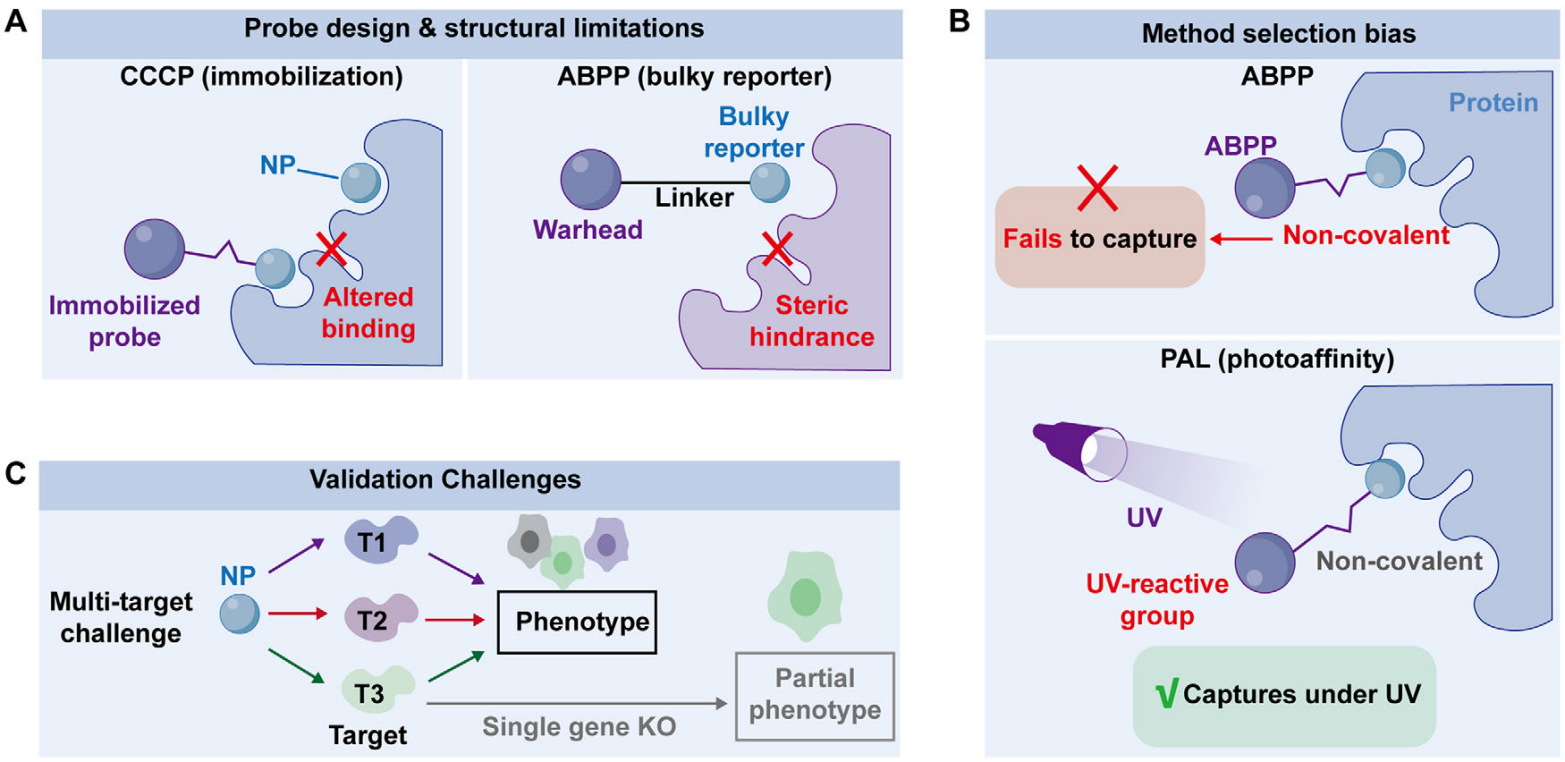
Limitations and biases in chemoproteomic target identification and validation. (A) Probe design and structural constraints. In CCCP‐based immobilization strategies, linkers or solid‐phase attachment may alter the native binding modes of natural products. In ABPP, bulky reporter groups can introduce steric hindrance, impairing authentic target engagement. (B) Methodological bias. Conventional ABPP is less sensitive to noncovalent interactions, whereas photoaffinity labeling enables the capture of transient or reversible interactions through ultraviolet (UV)‐activated photoreactive groups. (C) Validation challenges under polypharmacological mechanisms. Natural products frequently exert multitarget effects, and single‐gene knockout or single‐target perturbation may fail to fully recapitulate observed phenotypes. Therefore, hierarchical and multidimensional validation strategies are required.

The reproducibility of NP target identification is strongly influenced by chemical probe design and the inherent limitations of enrichment‐based strategies. CCCP provides a representative example. Since CCCP relies on sufficiently strong interactions between NPs and target proteins, its ability to capture weakly bound or low‐abundance targets is limited. In addition, nonspecific adsorption may generate false‐positives hits. More importantly, structural modifications of NPs for immobilization or linker introduction may alter their intrinsic biological activity, resulting in a bias toward capturing proteins that do not represent the true pharmacological targets of the parent compound. For example, by immobilizing total ginsenosides via oxidation at glycoside sites, researchers captured nearly 40 potential binding proteins. Further validation using biolayer interferometry and isothermal titration calorimetry confirmed the direct interaction between muscle‐type creatine kinase and 20(S)‐protopanaxadiol. This study demonstrates the feasibility of the immobilization strategy, while the broad‐spectrum capture profile also highlights the need for more stringent controls and hierarchical validation to distinguish direct targets from cobinding or nonspecific proteins [239] (Figure 8A).

In ABPP system, the size and chemical properties of reporter groups represent another source of variability. ABPP typically relies on the integration of a reactive warhead, linker, and reporter tag. Bulky tags, such as biotin or fluorophores, may weaken the biological activity of warhead, alter cellular distribution, or reduce affinity for genuine targets. In addition, conventional ABPP is relatively insensitive to noncovalent interactions, which may lead to divergent target profiles for the same NP across different studies. For example, researchers constructed a biotinylated probe based on raddeanin A and captured TAR DNA‐binding protein 43 (TDP‐43), providing a clear target clue for its mechanism related to immunogenic cell death. However, such covalent labeling‐dependent strategies also imply that if a NP primarily exerts its phenotypic effects through reversible noncovalent interactions, its relevant targets may be systematically overlooked [240].

Accordingly, expanding the ability to capture noncovalent interactions through PAL has become an important trend in recent years. Upon UV irradiation, PAL generates reactive intermediates that covalently crosslink with nearby amino acid residues, increasing the likelihood of identifying noncovalently bound NP–protein pairs [241]. For example, the diazirine photosensitizer probe okicamelliaside was shown to captures HSP90 in A549 cells after activation at 365 nm. By integrating molecular dynamics simulations and site‐directed mutagenesis, the study identified Glu47 as a critical hydrogen‐bonding site, highlighting the importance of an integrated “capture‐structure interpretation‐function validation” strategy for improving reproducibility [242]. Conversely, an opposite example also illustrates how method selection can decisively influence outcomes. Harringtonolide failed to capture targets using biotinylated probes in an ABPP format due to its reversible noncovalent binding properties. However, when a PAL–click chemistry composite probe was used, receptor for activated C kinase 1 was identified as its reversible noncovalent target. This finding underscores the importance of cross‐platform validation for avoiding methodological bias [243] (Figure 8B).

At the validation level, the multitarget and network‐level effects of NPs often mean that genetic validation of a single target can explain only part of the observed phenotype (Figure 8C). Meanwhile, stricter quantitative comparisons and competitive experimental designs are required to address unavoidable nonspecific binding during target capture. Quantitative chemical proteomics provides a critical solution [244]. In one study, spike‐in SILAC combined with a competitive binding gradient was used for target screening of zerumbone. Proteins showing decreased enrichment with increasing concentrations of the parent compound were identified as specific targets, ultimately yielding 62 significantly enriched proteins associated with apoptosis and survival processes. This approach illustrates how a quantifiable chain of evidence enhances the credibility of target screening [245].

### 7.2 Data Quality, Annotation, and Bias

At the data level, a major limitation is the frequent incompleteness of three essential components: chemical structure, experimental conditions, and identification results. In target capture experiments, the absence of standardized reporting for key parameters, such as probe activity fidelity, linker attachment site, elution conditions, and buffer systems, substantially reduces the reusability of result and limits cross‐study comparison. In chemical proteomics practice, nonspecific proteins, active metabolites, and genuine targets may be captured simultaneously. Without appropriate controls and quantitative strategies, experimental noise can easily be misinterpreted as meaningful signal. Moreover, the steric hindrance introduced by immobilized probes may alter binding behavior, resulting in false positives or omission of important targets. If such structural biases are not documented and transparently reported, they are difficult for subsequent studies to identify and correct [14, 246].

Bias is also reflected in system selection and result presentation. On the one hand, complex samples, such as herbal formulas or crude extracts, often generate large numbers of binding proteins in target capture experiments, thereby increasing the difficulty of false‐positive management during data interpretation. For example, researchers used photo‐crosslinked magnetic nanoparticles to capture targets from Jingfang Granules extract and identified 186 specific binding proteins in lung tissue [247]. Similarly, target identification experiments for Kaixin San extract identified 199 potential targets [248]. Such high target counts may reflect genuine multicomponent and multitarget properties at the and level, but they may also include numerous binding proteins unrelated to therapeutic efficacy. Therefore, the absence of rigorous control designs, confidence grading, and functional validation makes these datasets more difficult to reuse or incorporate into computational model training.

On the other hand, negative results and failed probes or experimental conditions are rarely documented systematically. For example, conventional ABPP is insensitive to noncovalent interactions. If a study reports only that no targets were captured without documenting whether the probe disrupted biological activity or whether the interaction might be noncovalent, it creates a data‐level bias through missing mechanistic information. The contrast of harringtonolide failing under ABPP conditions but succeeding with PAL–click chemistry precisely illustrates that negative data are critical for defining methodological boundaries and improving future method selection.

### 7.3 Need for Community Standards and Shared Resources

In NP target identification, the most direct consequence of lacking unified standards is the inconsistency of evidence thresholds used to define confirmed targets across studies. This inconsistency makes it difficult to compare candidate targets, integrate findings, and translate results into computational prediction or clinical development. For probe design, actionable minimum standards should be established. Probe modifications should avoid disrupting the activity of parent compound, and probes should demonstrate effective labeling capability, for example through in‐gel fluorescence, before MS‐based target identification [249]. Regarding control system, immobilization and enrichment strategies should include explicit negative controls and competition experiments, and protocols for handling nonspecific binding proteins should be incorporated into the methodological framework [250].

Quantitative standardization is essential for improving inter‐laboratory comparability. SILAC enables stable isotope labeling at the cellular level, and merging samples after enrichment can reduce sample processing errors and improve reproducibility. The identification of inosine monophosphate dehydrogenase 2 (IMPDH2) as a key target of sappanone A using SILAC pull‐down demonstrates the feasibility of combining standardized quantitative workflow with specific target identification [244]. For samples in which cellular labeling is not feasible, such as tissues or natural communities, post‐labeling strategies such as iTRAQ can serve as alternatives. These approaches can be applied to target screening and subsequent functional validation of NP‐derived probes, including clickable andrographolide probes.

At the resource level, there is a pressing need for interoperable data units that integrate structural information, experimental conditions, control designs, identification results, and evidence levels. Such data units would enable other researchers to reproduce, reanalyze, and computationally integrate published findings. Particular emphasis should be placed on constructing benchmark datasets that include negative results. For example, systematic documentation of cases in which a probe failing to capture targets under ABPP but succeeds under PAL–click chemistry would substantially improve method selection and model training. In addition, standardization of spatial and temporal resolution as well as in vivo validation should be incorporated into future roadmap. These efforts should include subcellular target localization, enhanced spatiotemporal resolution, organoid systems, and humanized animal models. The ultimate goal is to transform targets that are capturable in vitro into targets that are verifiable in physiological environments [250].

## 8 Conclusions and Future Perspectives

In summary, the core value of NP target discovery lies not merely in identifying potential binding proteins, but in translating complex phenotypes‐associated molecular networks into verifiable, interpretable, and translatable mechanistic frameworks. This field is therefore shifting from competition among individual technologies toward a broader emphasis on evidence organization and research design logic.

From a methodological perspective, experimental and computational techniques play distinct but complementary roles in resolving uncertainty across different application scenarios. Experimental measurements provide direct insights into NP‐target interactions and MOA within complex biological contexts, whereas computational tools can rapidly narrow candidate target spaces with lower costs and higher throughput in high‐dimensional biological and chemical spaces. Although the parallel use of both approaches is valuable, the more critical task is to organize them into a reusable integrated strategy. In such workflows, computational outputs guide the prioritization of experimental validation, while experimental results verify candidate targets and inform subsequent computational iterations. This enables target discovery to evolve into a continuously convergent closed‐loop process.

At the same time, the main reliability bottleneck in target identification usually arises less from the lack of advanced instruments or AI models than from poorly defined methodological boundaries. Systematic biases may originate from probe modifications, enrichment strategies, biological system selection, and control design. Inadequate reporting of essential experimental details makes results difficult to reproduce, integrate, reuse for model training, or compare cross methods. Therefore, future development should prioritize reproducibility, comparability, quantifiability, and traceability as goals that are as important as technological innovation.

Future breakthroughs may occur across three major dimensions. First, at the level of technology development, more suitable methods are needed for emerging NP modalities. These methods should more efficiently capture targets characterized by weak affinity, transient interactions, and limited druggability, while enabling higher‐resolution binding site and structural analysis without compromising the native activity of the parent compound. Second, at the level of model construction, AI technologies should be integrated with validation systems that move beyond simplified single‐cell models toward models that more closely mimic human disease states and clinically relevant readouts. Third, at the precision medicine level, target identification outcomes should be linked to quantifiable biomarkers, patient stratification strategies, and clinically actionable endpoints. This linkage would allow mechanistic evidence to inform indication selection, biomarker‐driven development, and rational combination therapy design, thereby translating the network effects of NPs into actionable drug development pathways.

Overall, this review does not emphasize the superiority of any single methodology. Instead, it proposes more generalizable research models and future directions for NP target discovery. As synergistic strategies integrating iterative experimentation and computation continue to mature, NP target discovery is expected to evolve from experience‐dependent mechanism exploration into a sustainable, continuously improvable, and systematic paradigm. This evolution should more efficiently support the discovery of novel therapeutic targets and the translation of NPs into new medicines.

## Author Contributions

Qiyuan Pan: literature collection and organization and manuscript writing and revision. Xiao Yuan: figure preparation and manuscript revision. Jinmei Jin, Xin Luan, Cheng Luo, and Hongzhuan Chen: conceptualization and revision of the manuscript. Weidong Zhang and Hao Zhang: conception of the manuscript and drafting the outline and manuscript revision. All authors have read, discussed, and approved the final manuscript.

## Ethics Statement

The authors have nothing to report.

## Conflicts of Interest

The authors declare no conflicts of interest.

## Data Availability

The authors have nothing to report.
